# Supplementary figures and images for: Host Protein Kinase C⍺: The novel Mitogen Activated Protein Kinase (MAPK) specific scaffold regulating nuclear export of influenza virus ribonucleoprotein complexes
Source: PLoS Pathog. 2025 Dec 31;21(12):e1013841. doi: 10.1371/journal.ppat.1013841 (PMC12788653; doi:10.1371/journal.ppat.1013841)

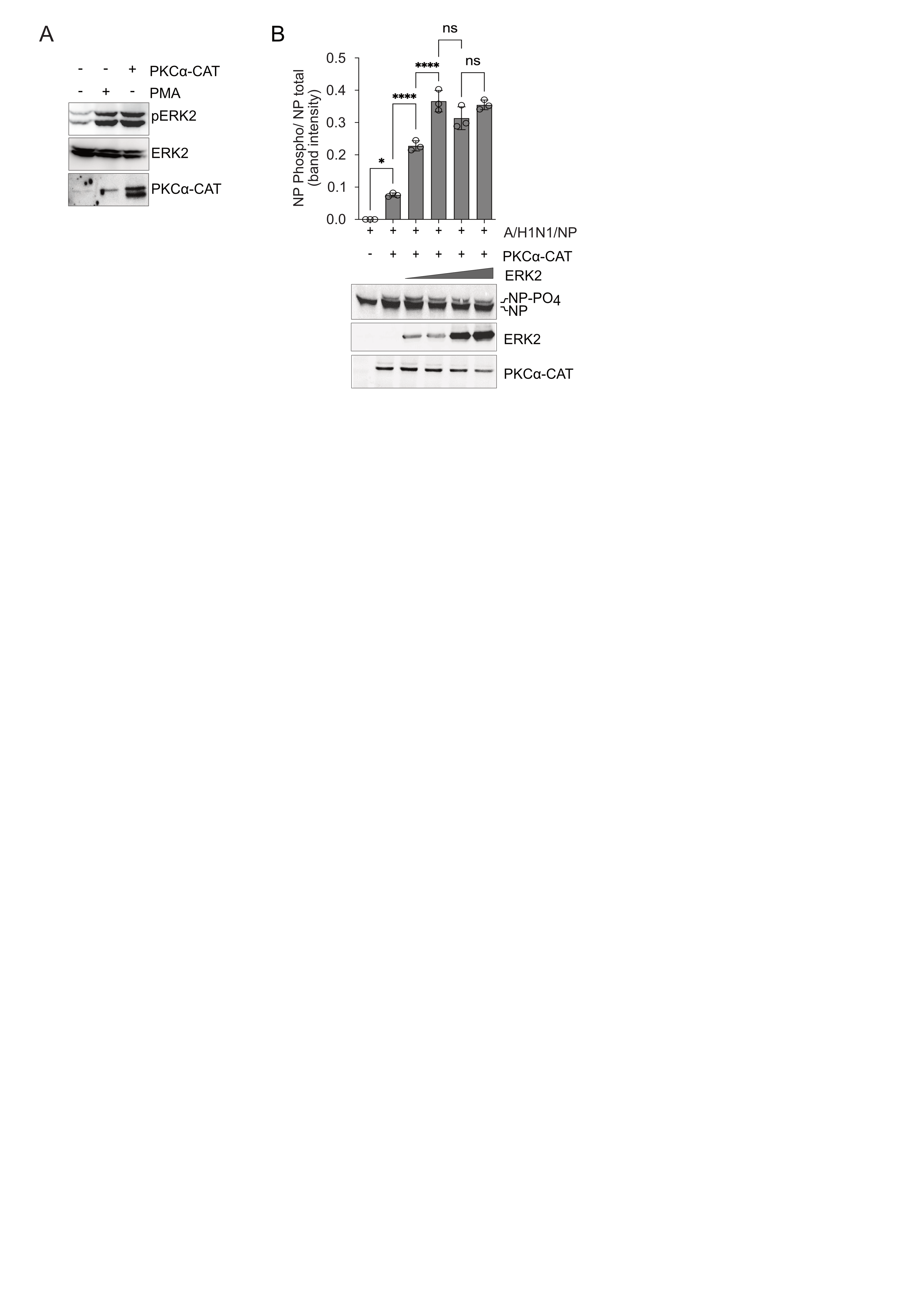

Supplement: S1 Fig — (B) Cells overexpressing NP-V5 with PKCα-CAT along with increasing concentration of ERK2-FLAG were analyzed by western blot analysis using anti-V5 antibody. Band intensities of hyperphosphorylated and unphosphorylated NP were assessed from three independent experiments are plotted using image J software. (TIFF) [file ppat.1013841.s001.tiff]

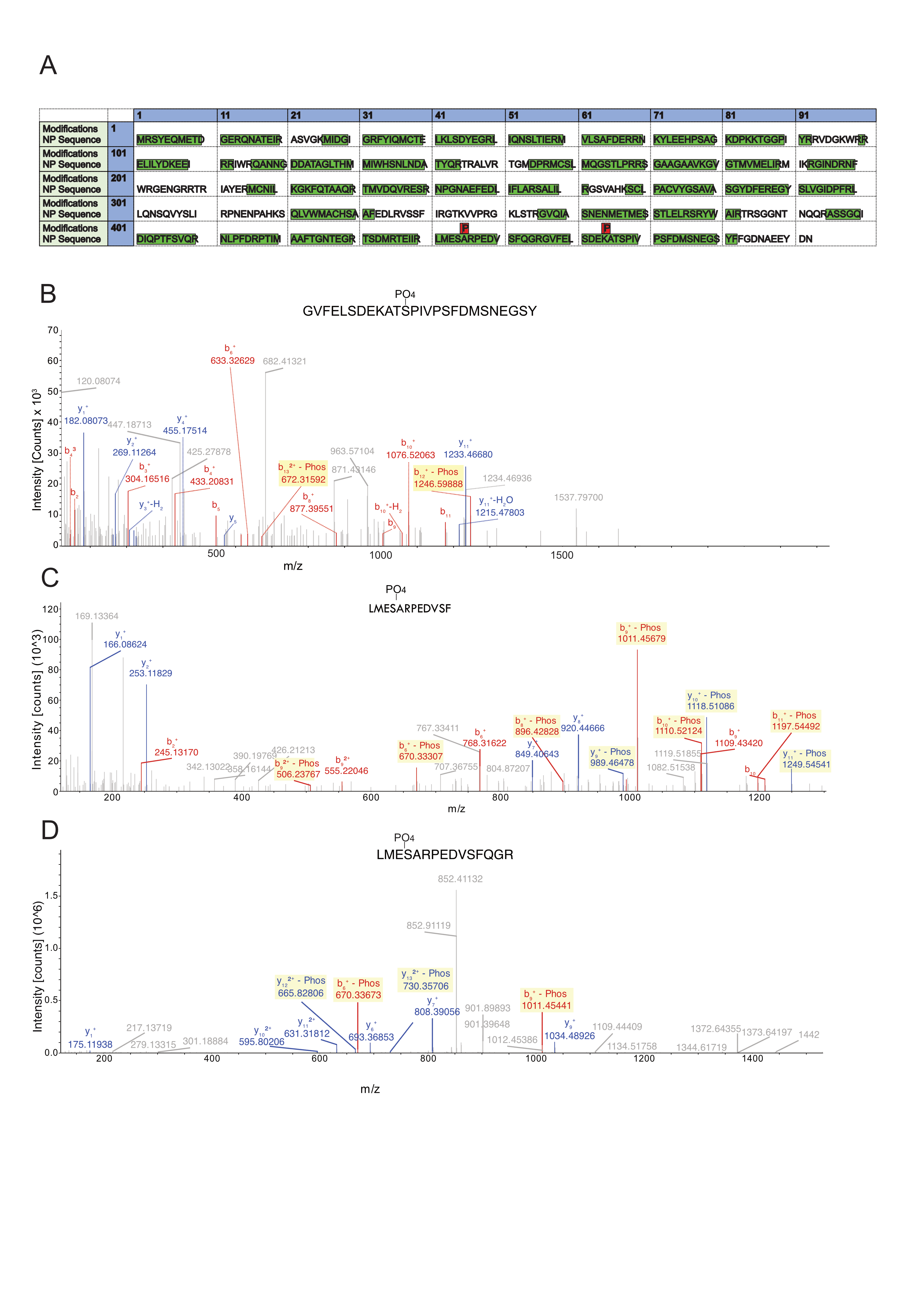

Supplement: S2 Fig — (A) Table showing peptide coverage for NP with phosphorylation of specific amino acid residues. (B-D) showing chromatograms of the phsopho-peptides subjected to CID fragmentation. Y and B ions are labelled in blue and red respectively. (TIFF) [file ppat.1013841.s002.tiff]

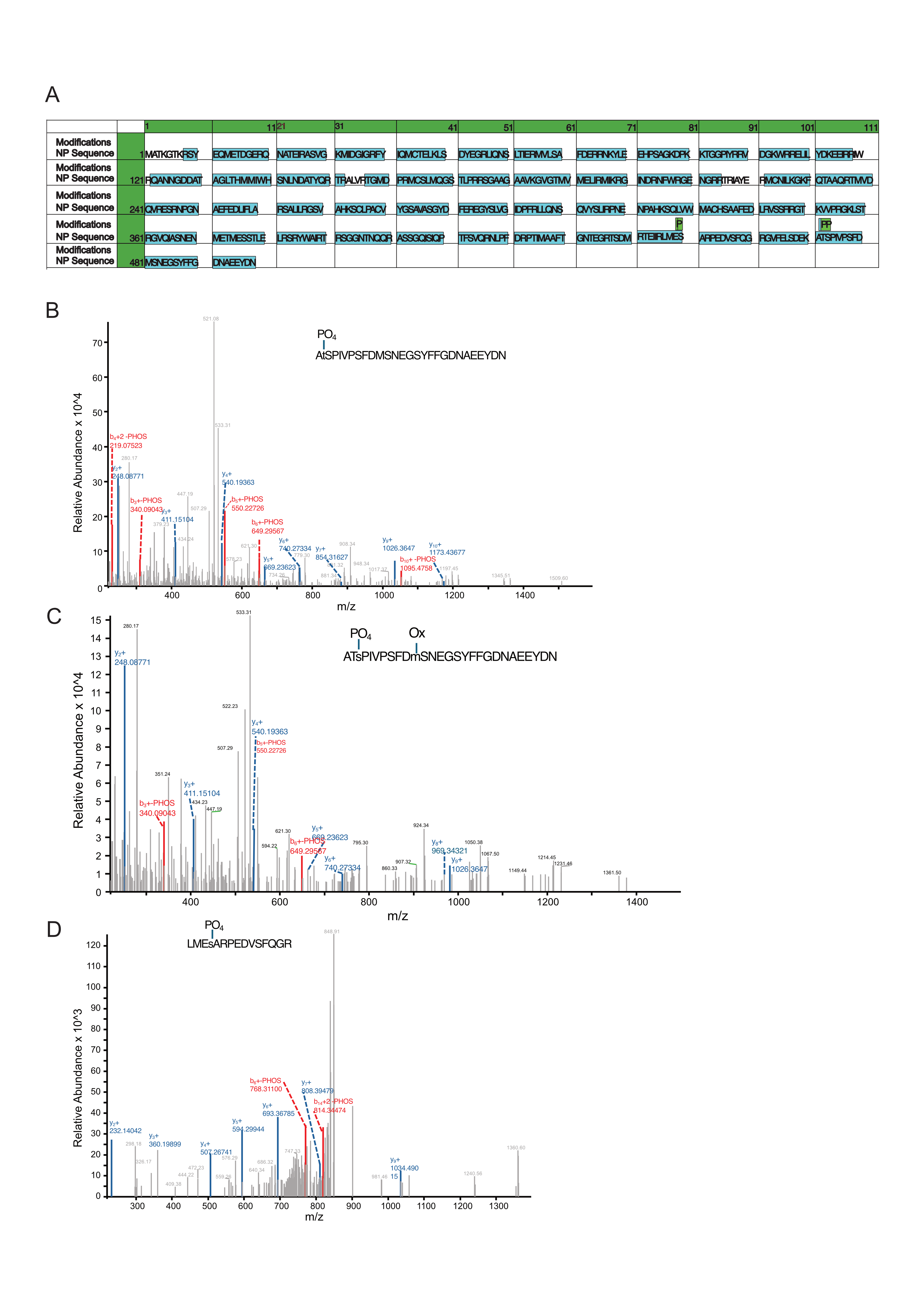

Supplement: S3 Fig — (A) Table showing peptide coverage for NP with phosphorylation of specific amino acid residues. (B-D) showing chromatograms of the phsopho-peptides subjected to CID fragmentation. Y and B ions are labelled in blue and red respectively. (TIFF) [file ppat.1013841.s003.tiff]

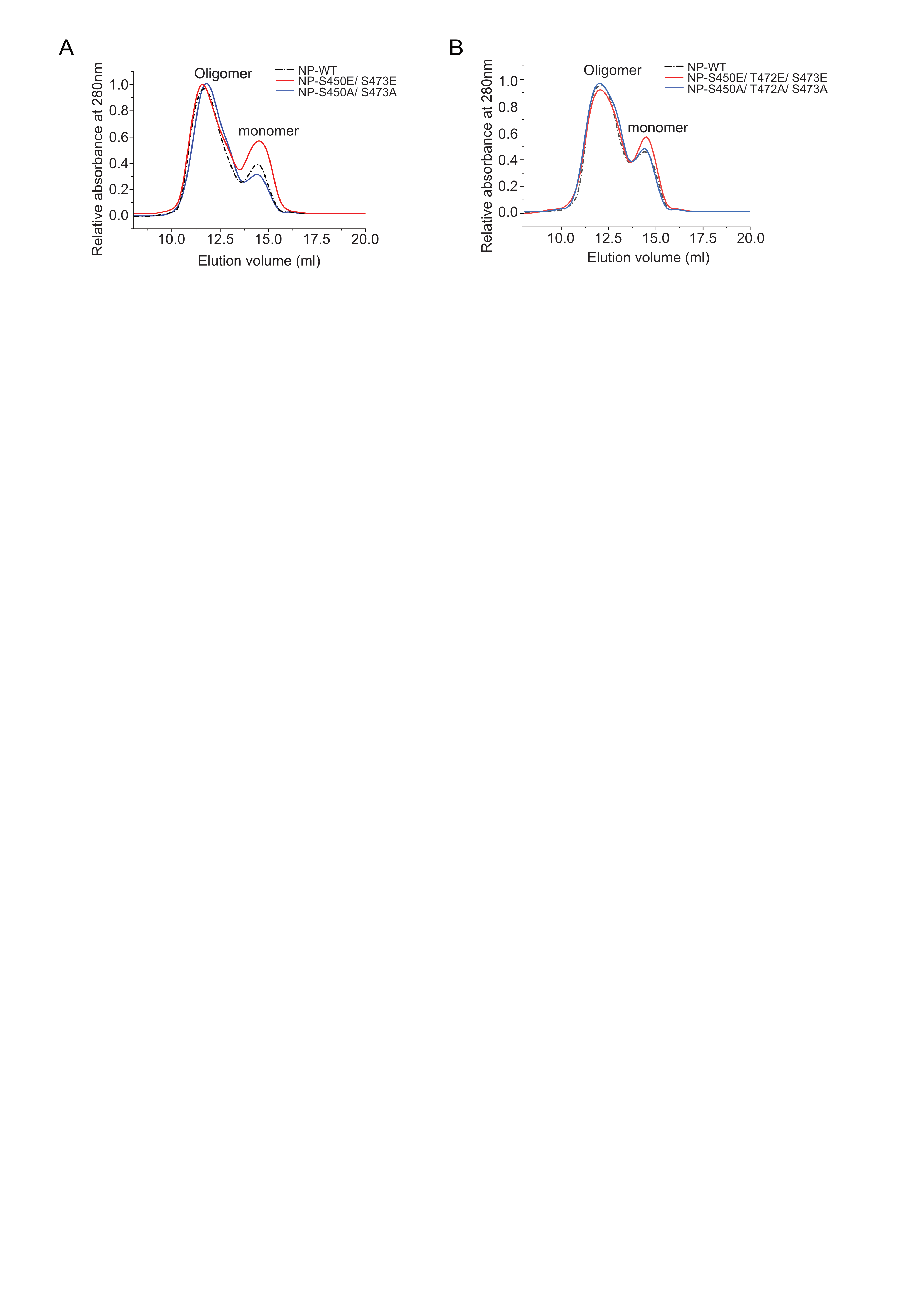

Supplement: S4 Fig — (TIFF) [file ppat.1013841.s004.tiff]

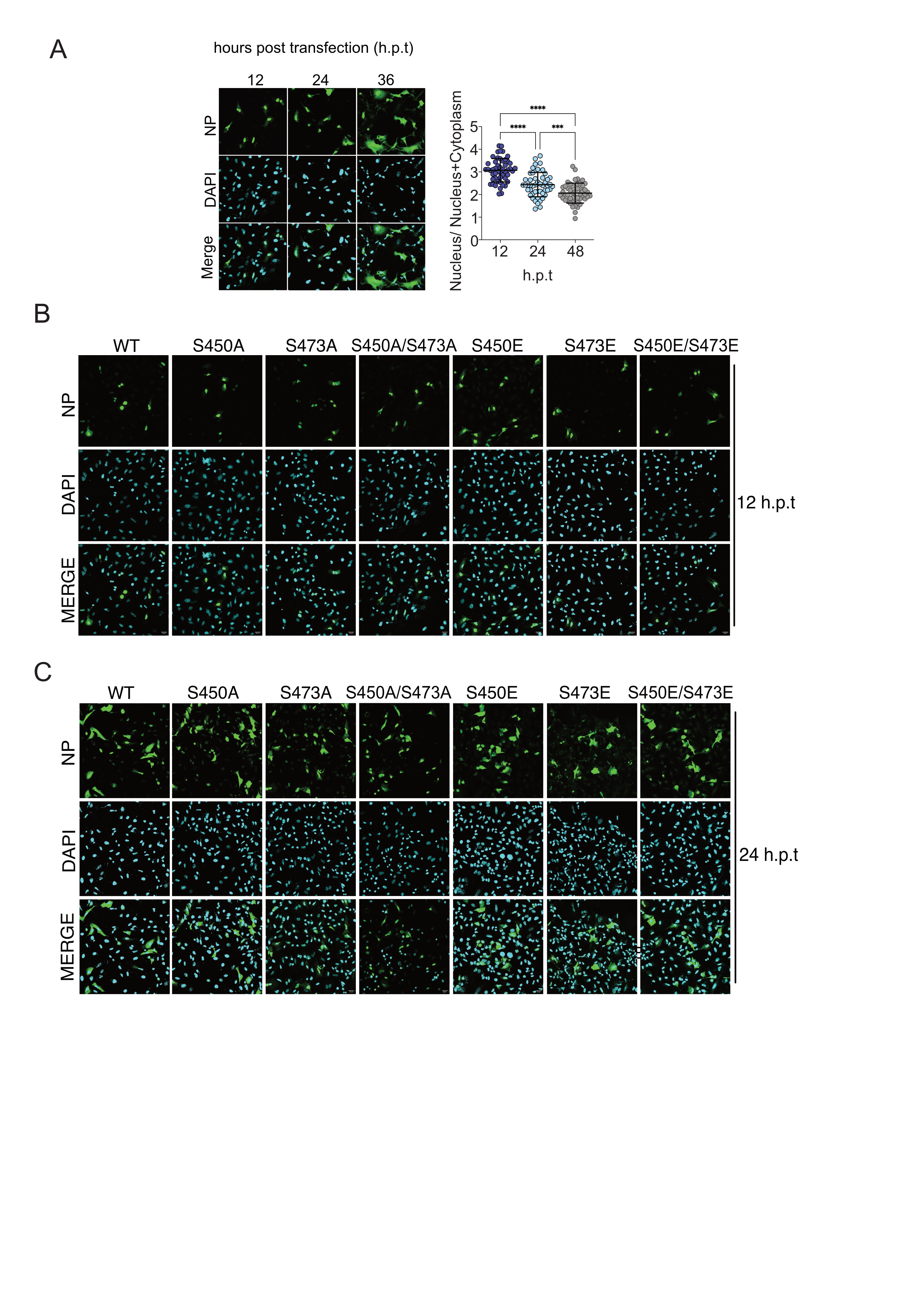

Supplement: S5 Fig — Cells were imaged using confocal microscope. (TIFF) [file ppat.1013841.s005.tiff]

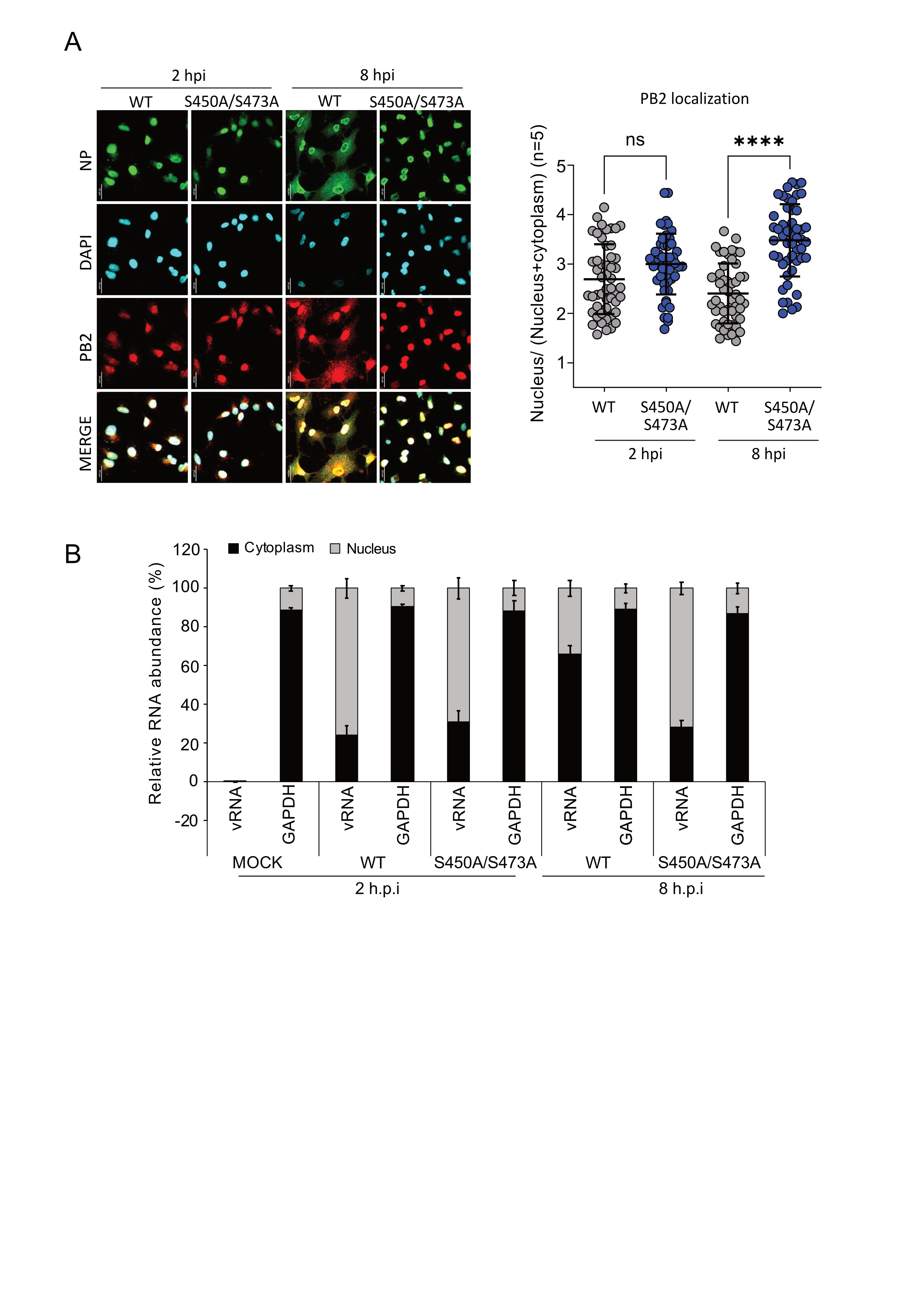

Supplement: S6 Fig — RNPs were visualised at 2 and 8 hpi by staining with NP and PB2 specific antibodies. Image analysis: 50 cells from 5 different fields were analysed using image J software to present nuclear-cytoplasmic distributions of NP. Each image is a representative of three independent biological replicates. Two way Anova was used to measure the statistical significance between the individual sets with P value (ns > 0.05; *P ≤ 0.05; **P ≤ 0.01; ***P ≤ 0.001). (B) A549 cells infected with WT or S450A/ S473A mutant viruses (MOI: 2) were harvested at 2 and 8 hpi followed by nuclear-cytoplasmic fractionation and total RNA isolation. qRT-PCR was performed to analyze the abundance of viral RNA (vRNA) and GAPDH in the subcellular compartments through normalization against U2 snRNA and 12s rRNA as nuclear and cytoplasmic reference genes. (TIFF) [file ppat.1013841.s006.tiff]

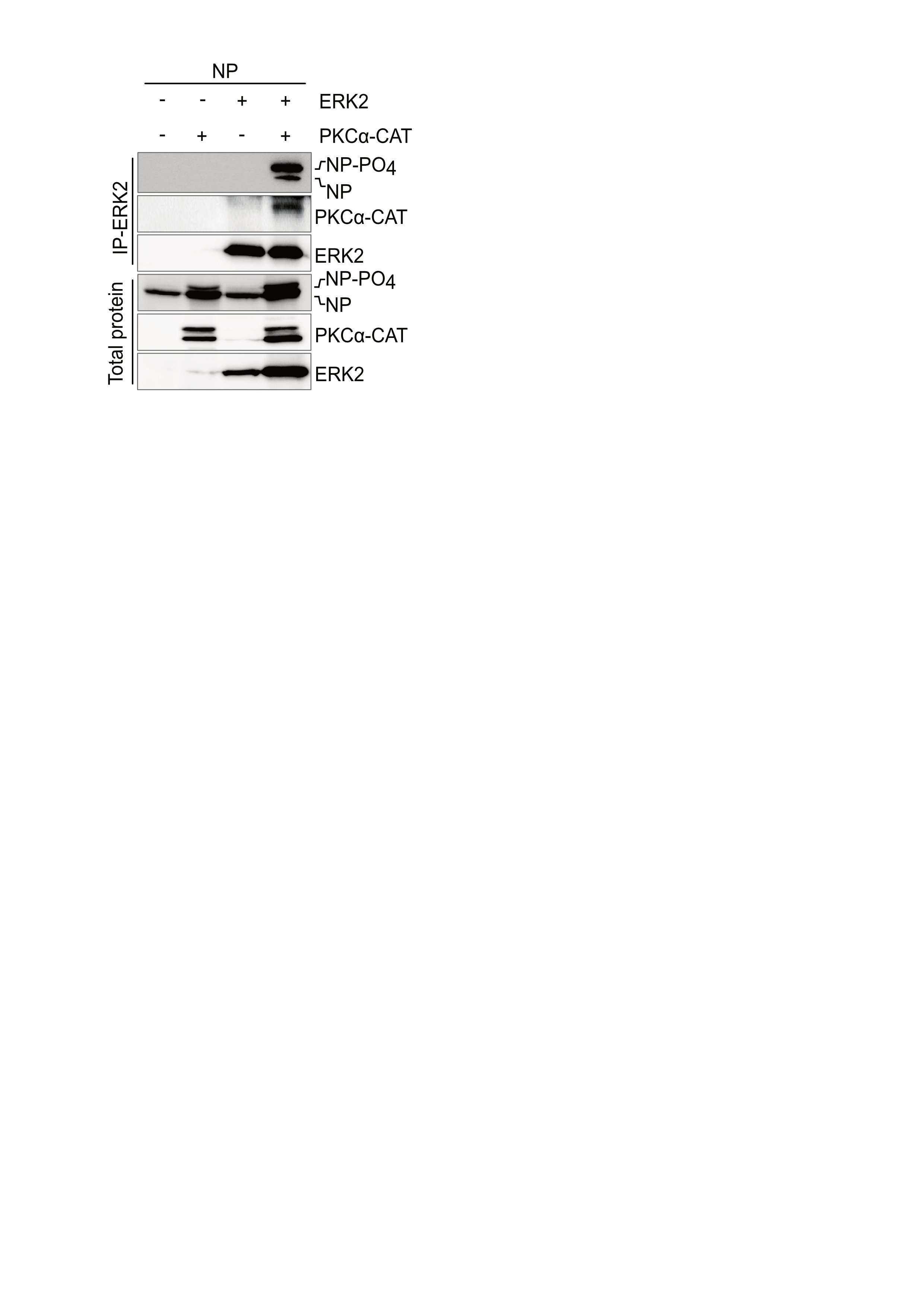

Supplement: S7 Fig — HEK293T cells were transiently transfected to overexpress NP-V5 either alone or in the context of ERK2-FLAG and PKCα-CAT-HA overexpression and immunoprecipitated using FLAG antibody. Co-precipitation of NP was observed using V5 antibody. Co-precipitation of ERK2 was observed using FLAG and HA (PKCα) antibody. (TIFF) [file ppat.1013841.s007.tiff]

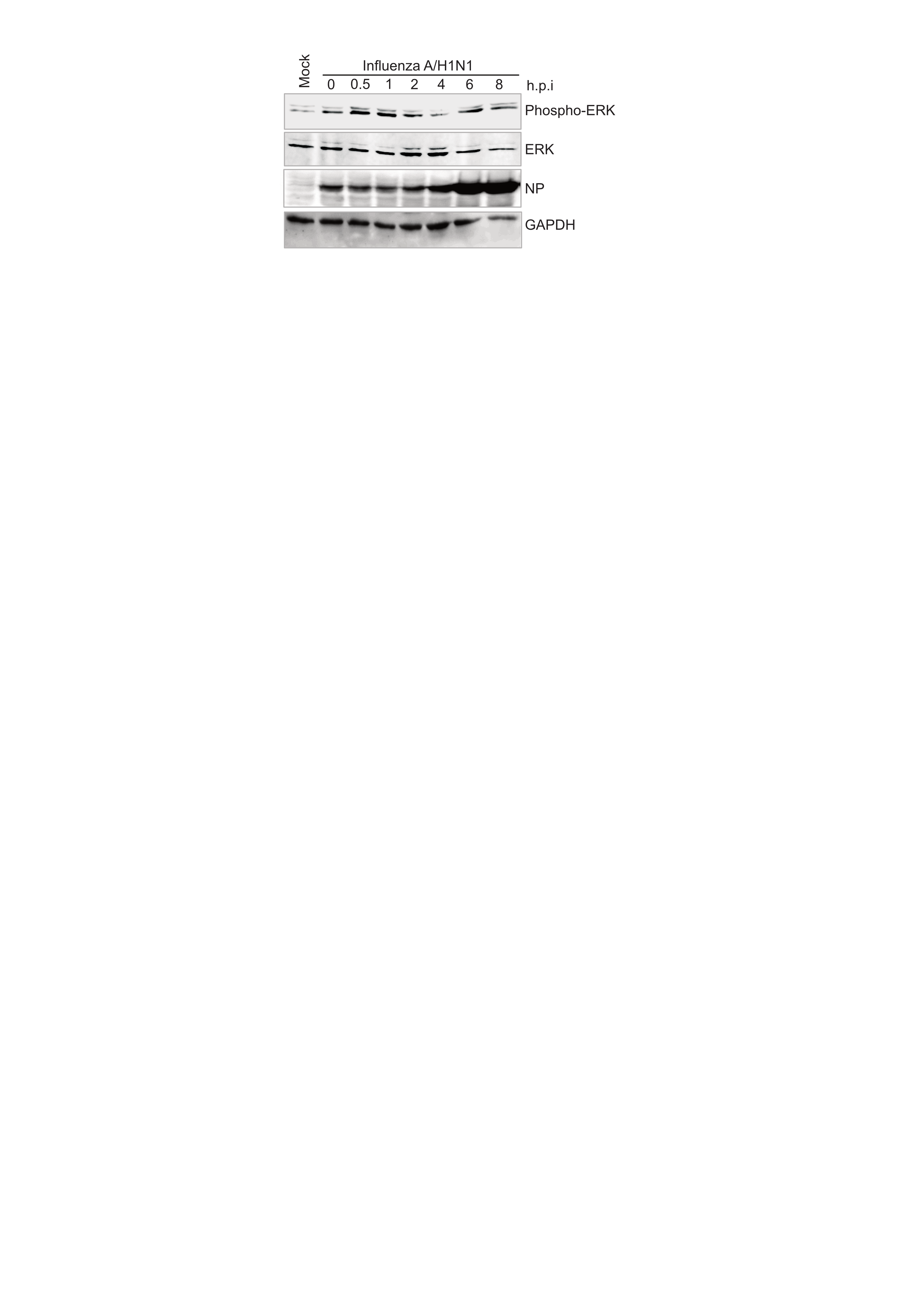

Supplement: S8 Fig — ERK2 activation was monitored using phospho-ERK antibody. NP served as infection marker while GAPDH as loading control. (TIFF) [file ppat.1013841.s008.tiff]

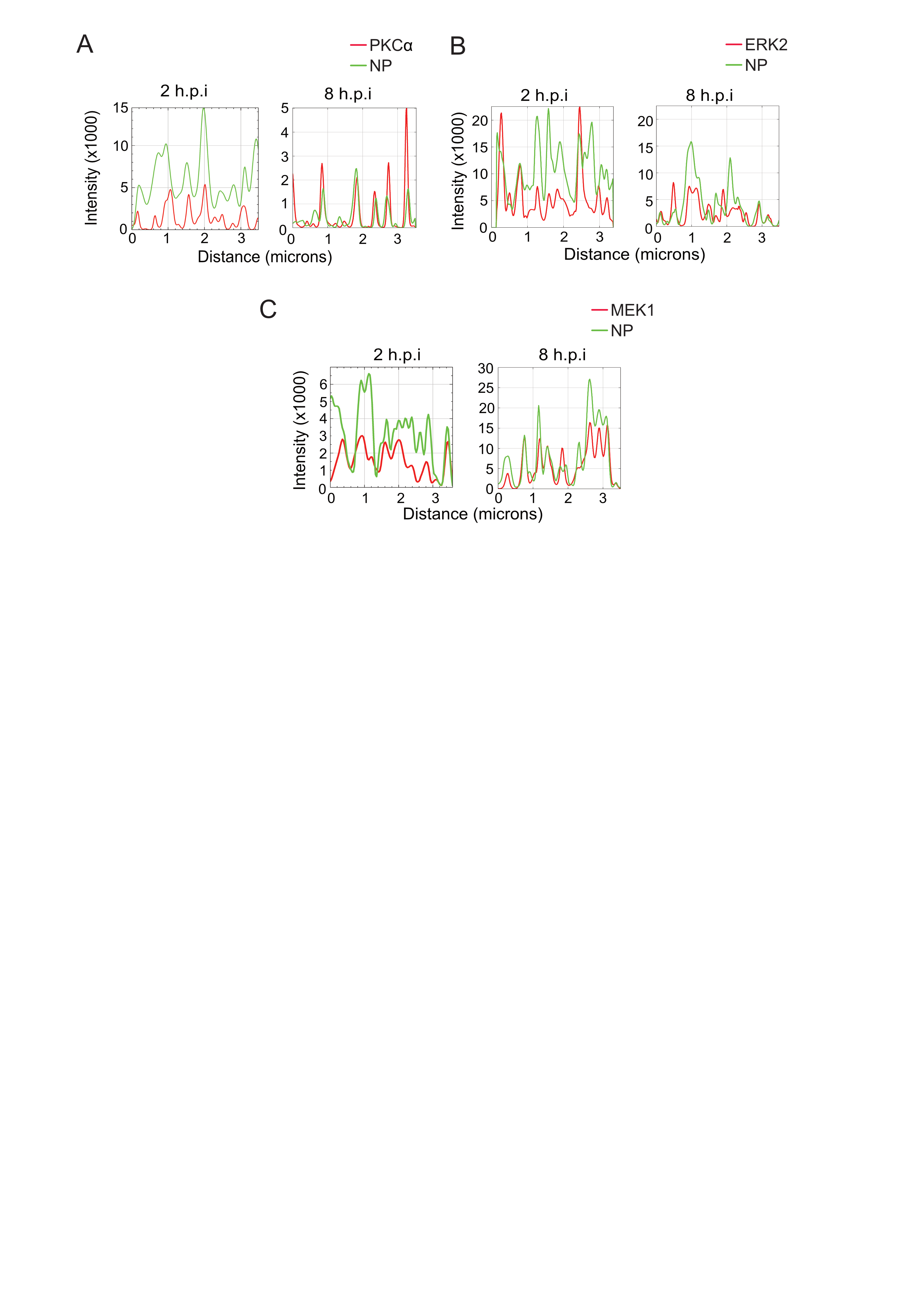

Supplement: S9 Fig — (TIFF) [file ppat.1013841.s009.tiff]

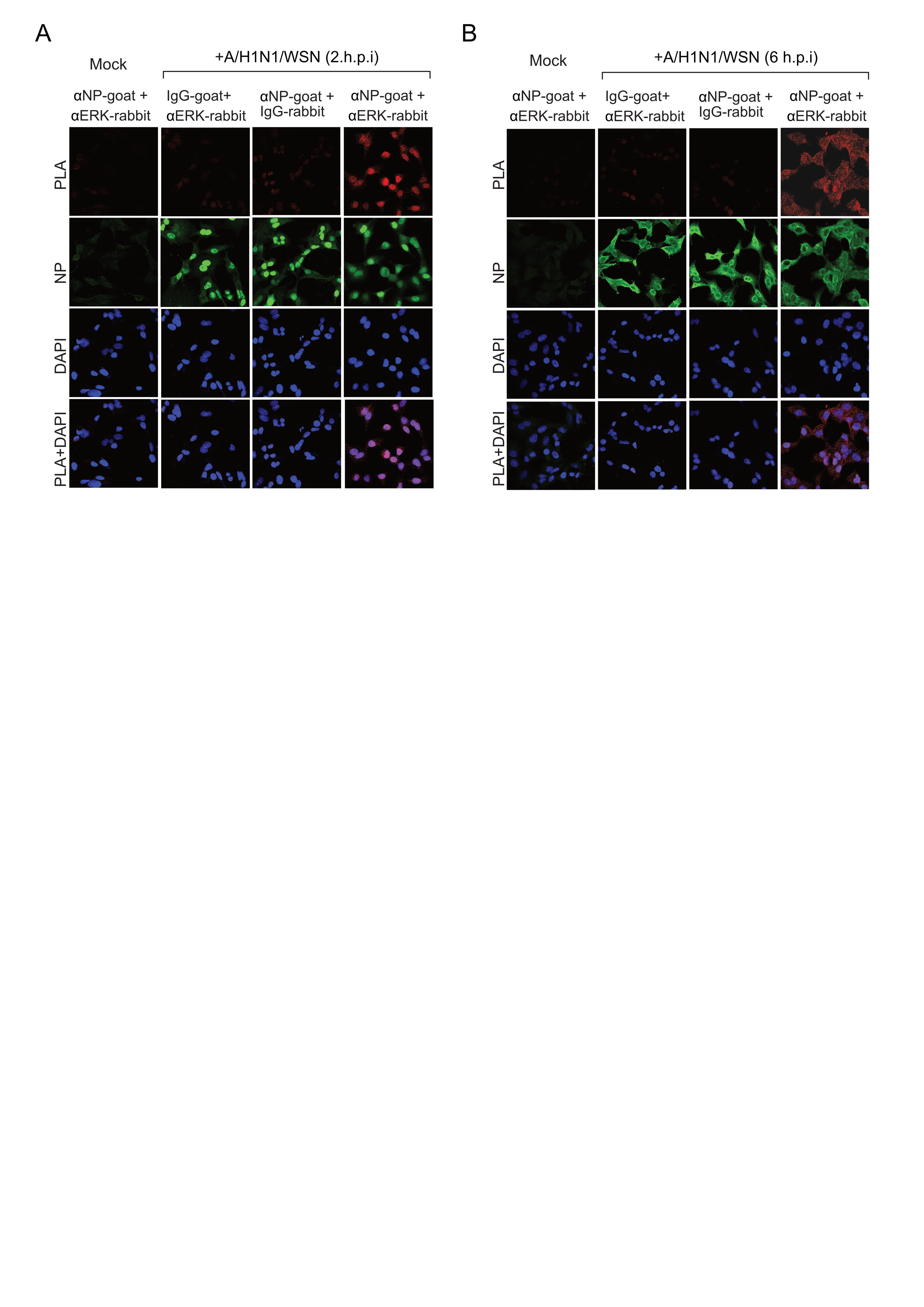

Supplement: S10 Fig — Post PLA, cells are stained with anti-NP antibody (mouse) and imaged. Quantitative analysis of the PLA dots are presented in main text Fig 8D-8F. (TIFF) [file ppat.1013841.s010.tiff]

**S1 Table.** Ion series for the peptide - LMESARPEDVSF


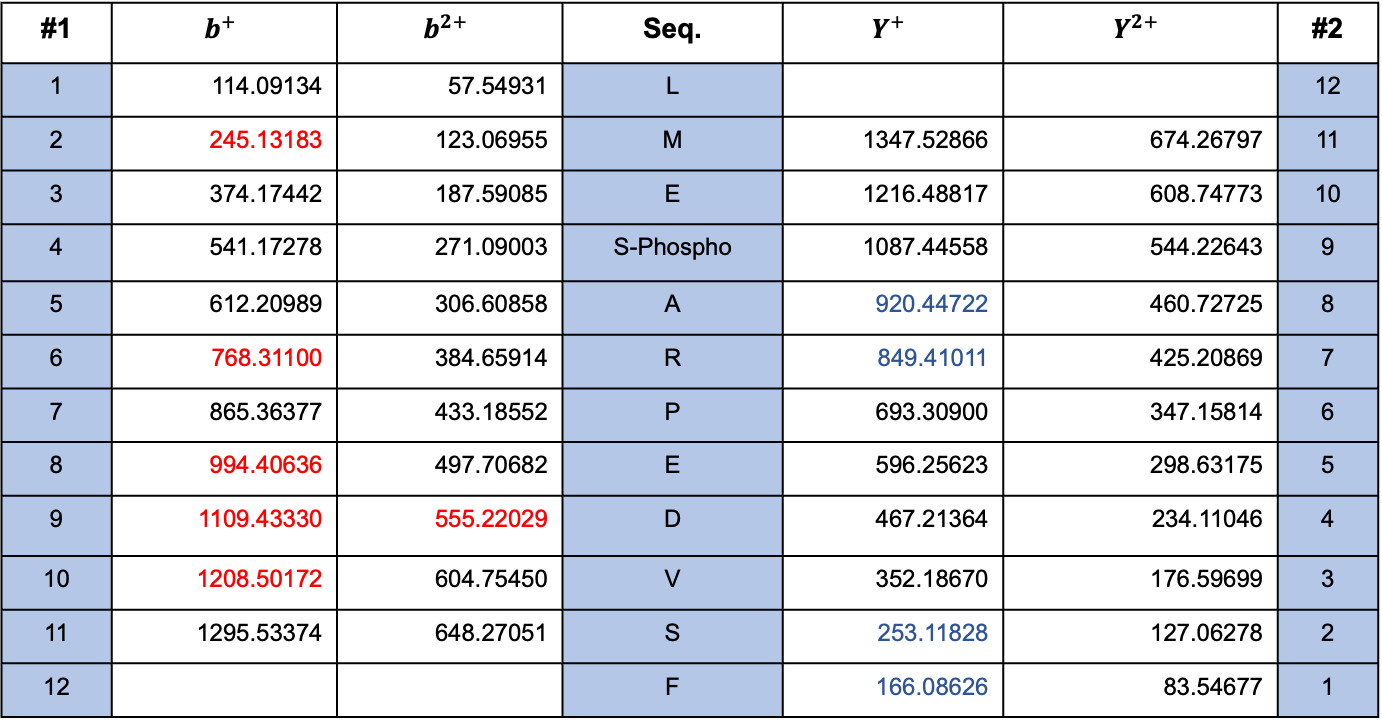

Supplement: S1 Table — (DOCX) [file ppat.1013841.s011.docx]

**S2 Table.** Ion series for the peptide: LMESARPEDVSFQGR


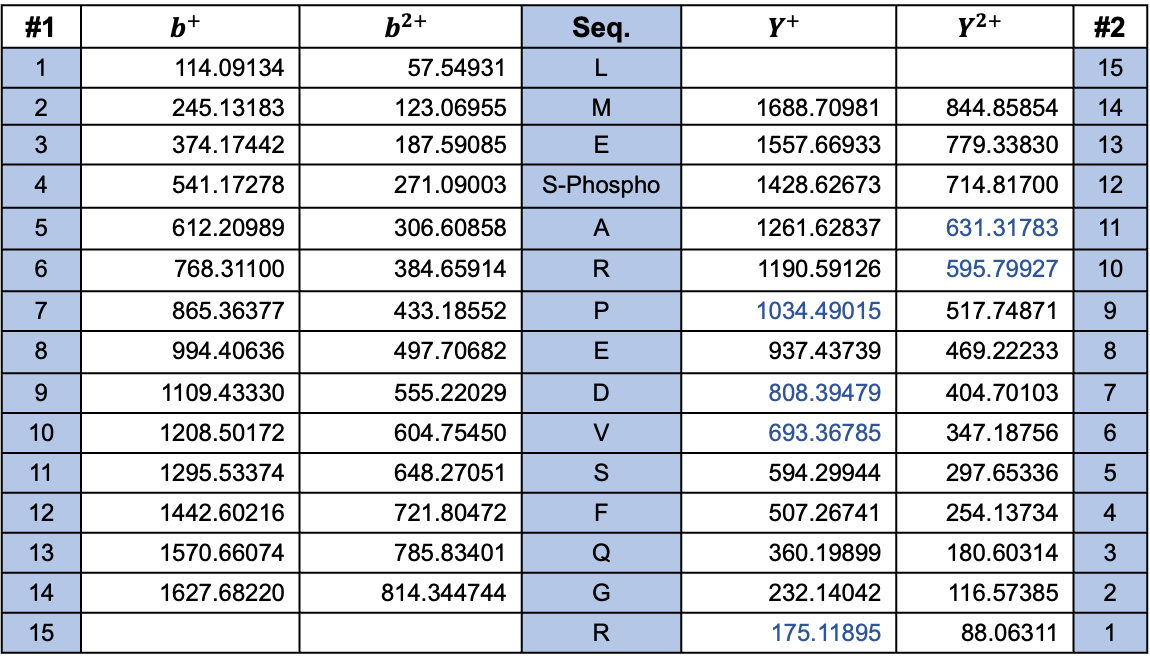

Supplement: S2 Table — (DOCX) [file ppat.1013841.s012.docx]

**S3 Table.** Ion series for the peptide: GVFELSDEKATSPIVPSFDMSNEGSY

**
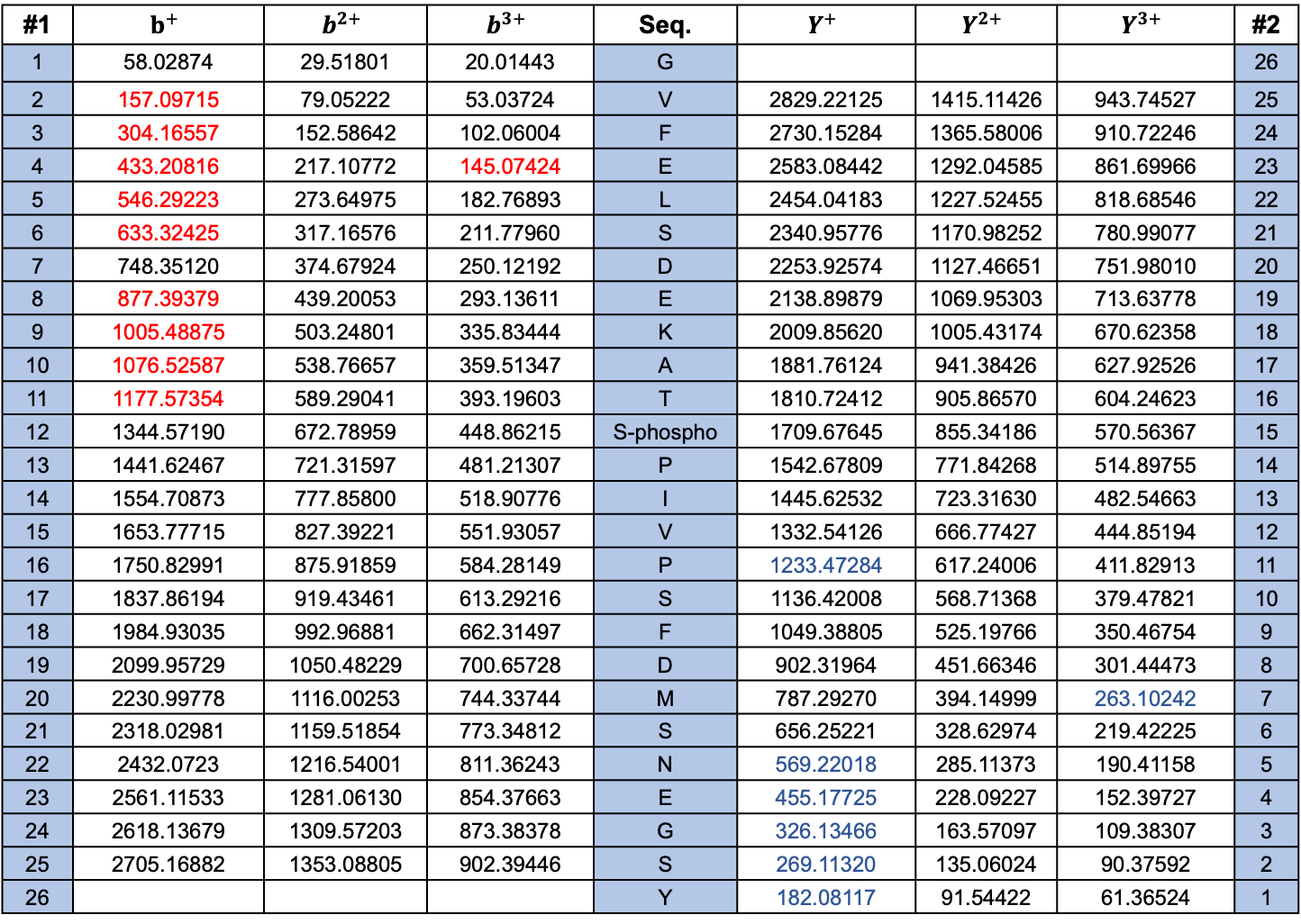
**

Supplement: S3 Table — (DOCX) [file ppat.1013841.s013.docx]

**S5 Table:** Ion series for the peptide: ATSPIVPSFDMSNEGSYFFGDNAEEYDN, S3-Phospho (79.96633 Da)


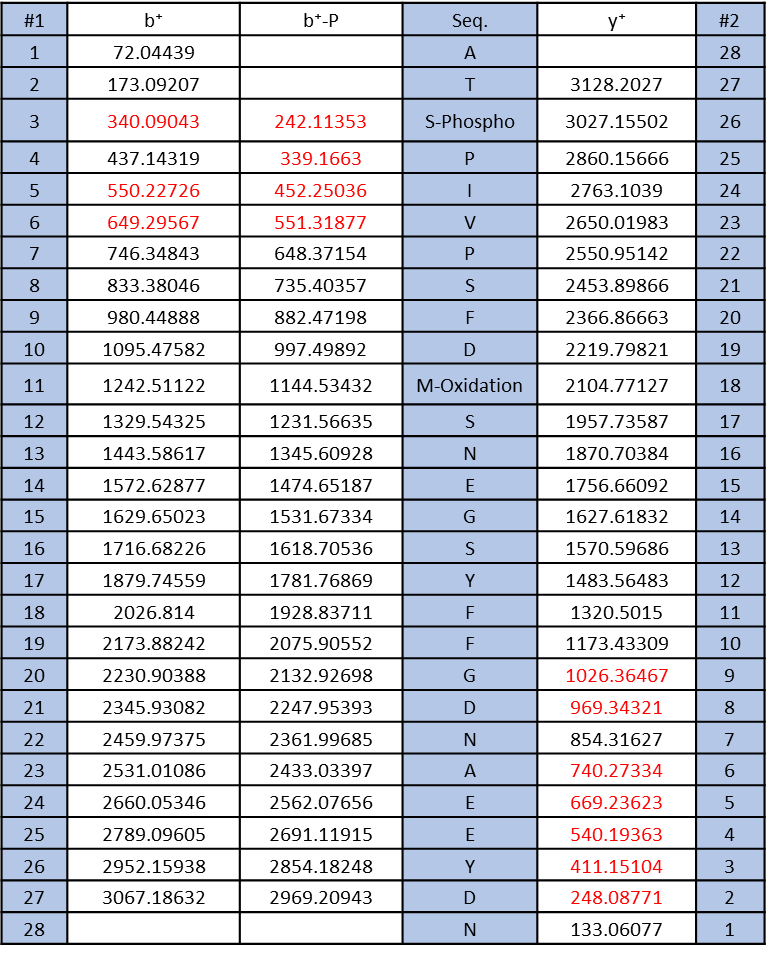

Supplement: S5 Table — (DOCX) [file ppat.1013841.s015.docx]

**S6 Table:** Ion series for the peptide: LMESARPEDVSFQGR, S4-Phospho (79.96633 Da)


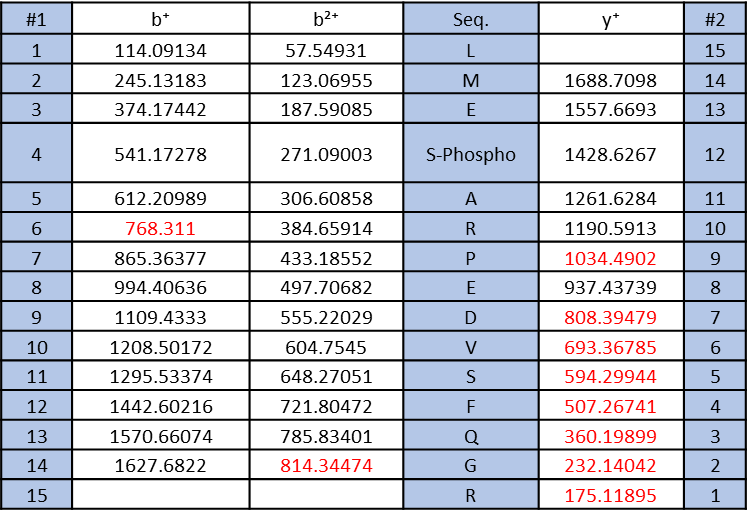

Supplement: S6 Table — (DOCX) [file ppat.1013841.s016.docx]

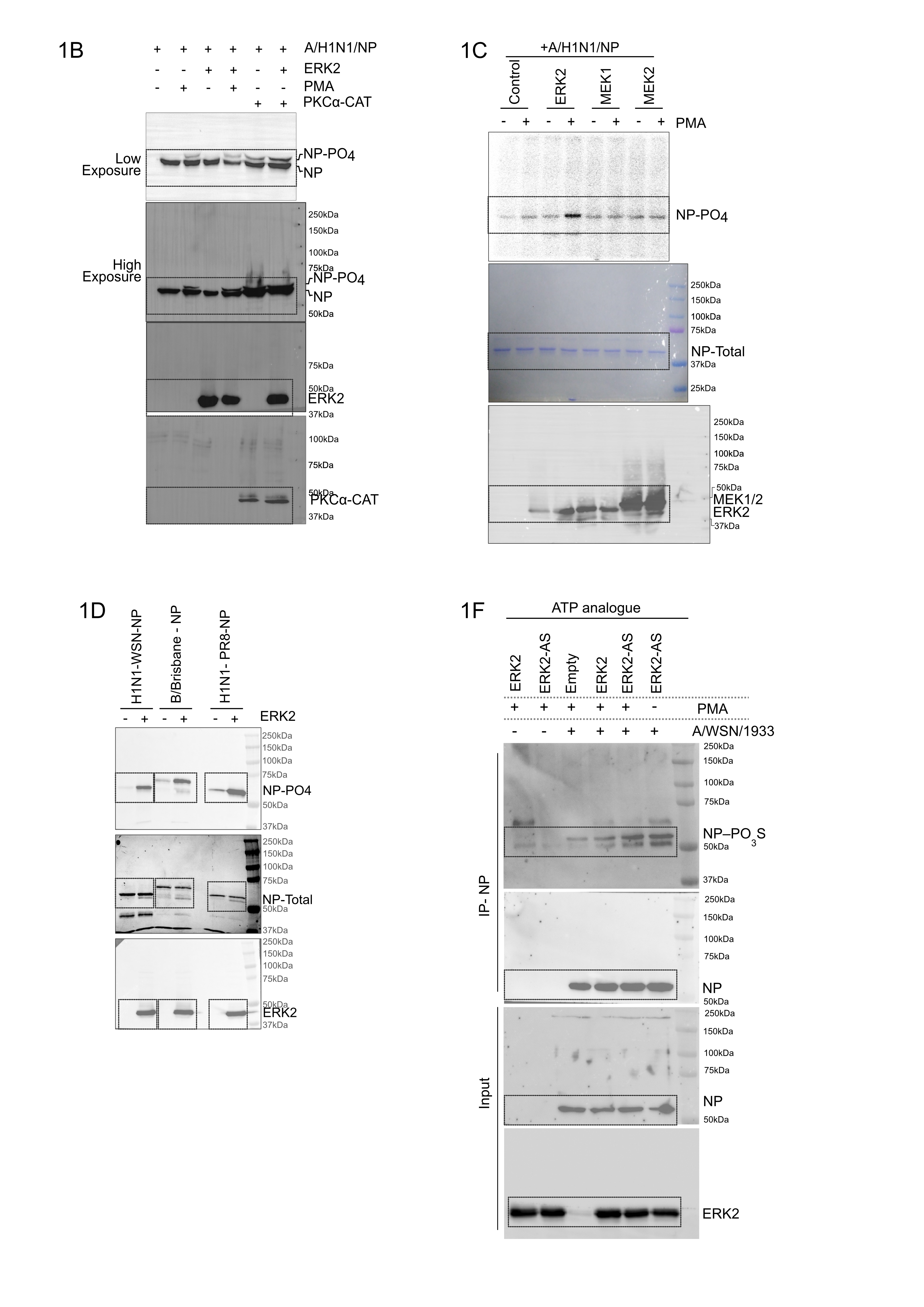


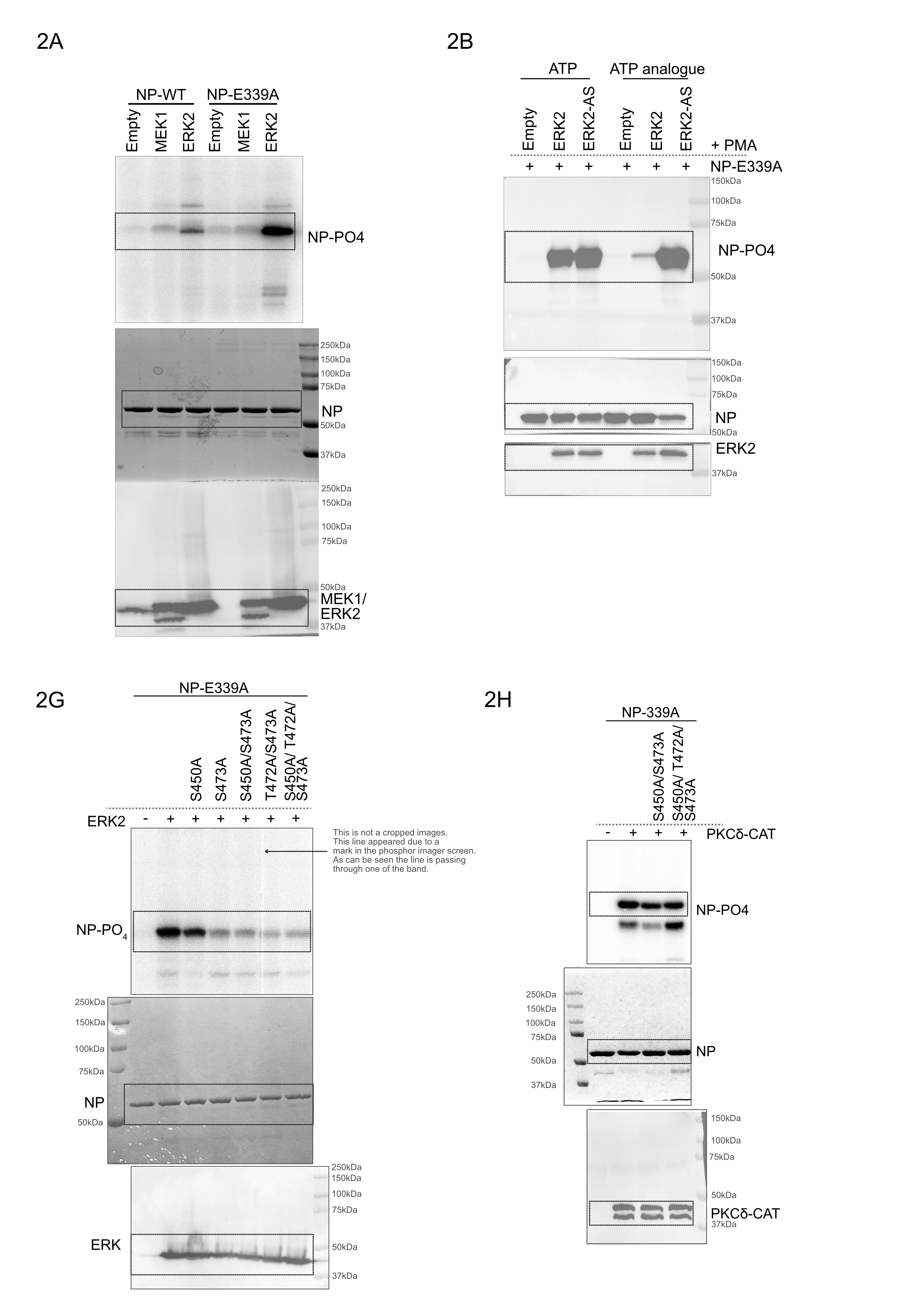


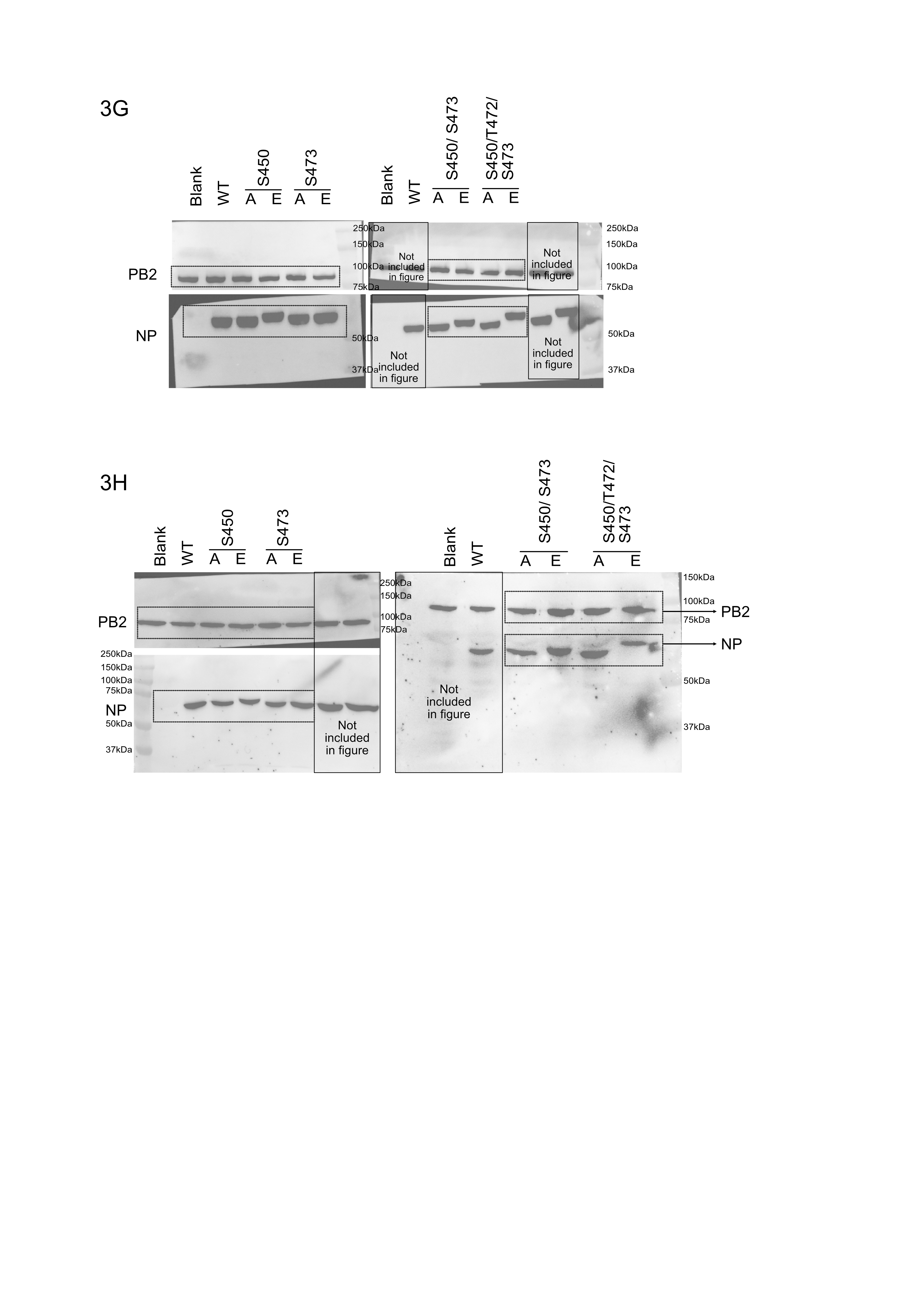


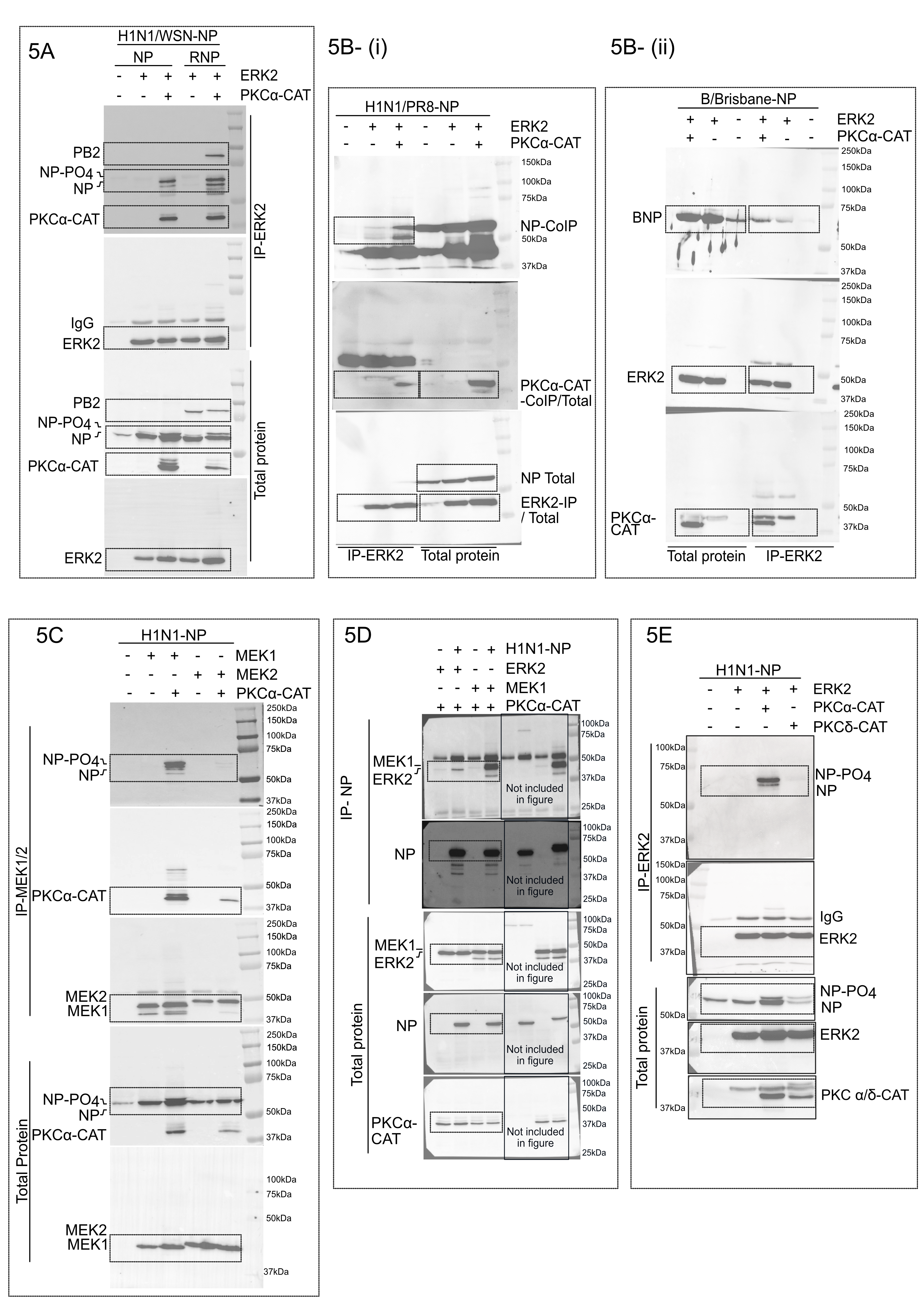


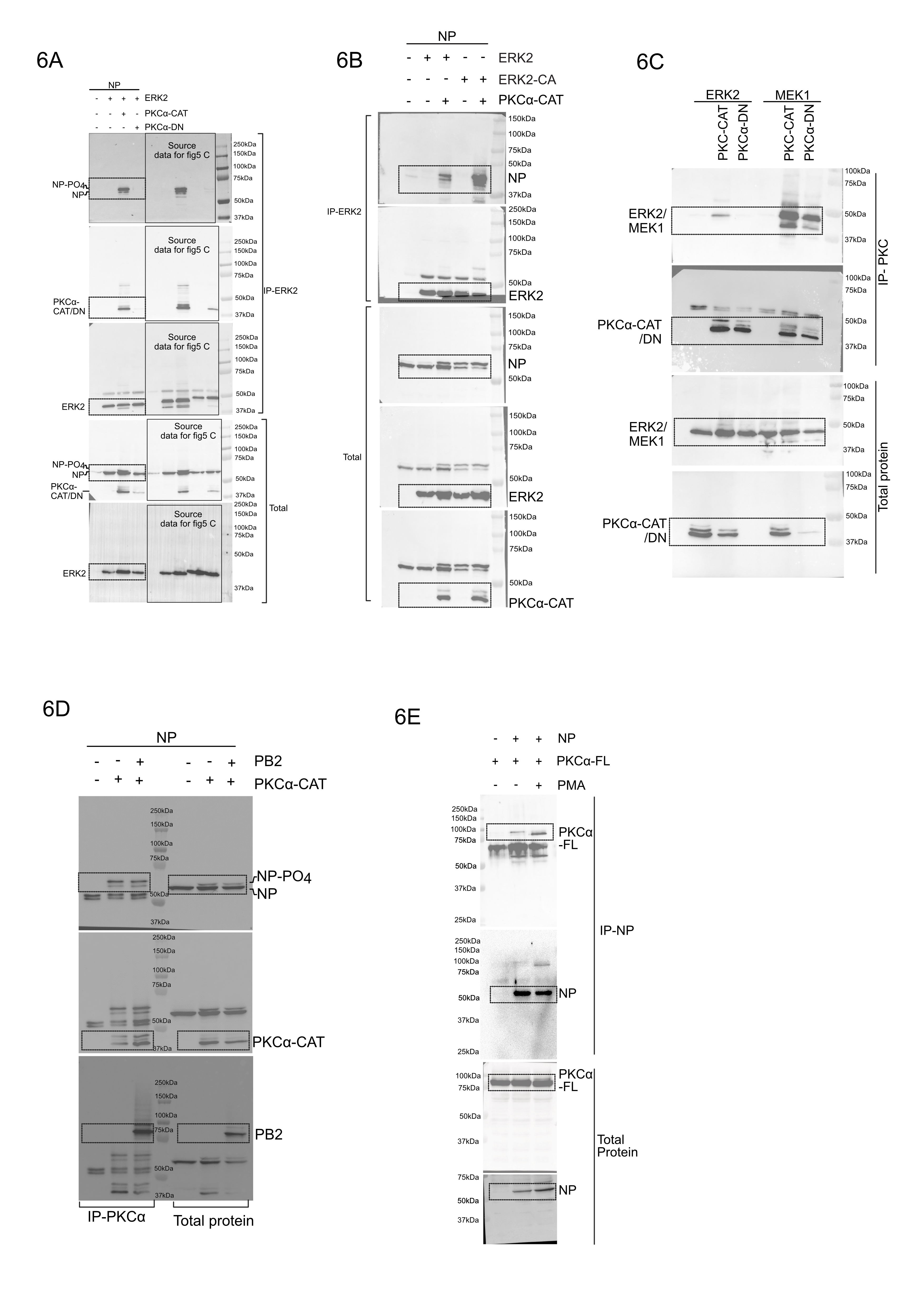


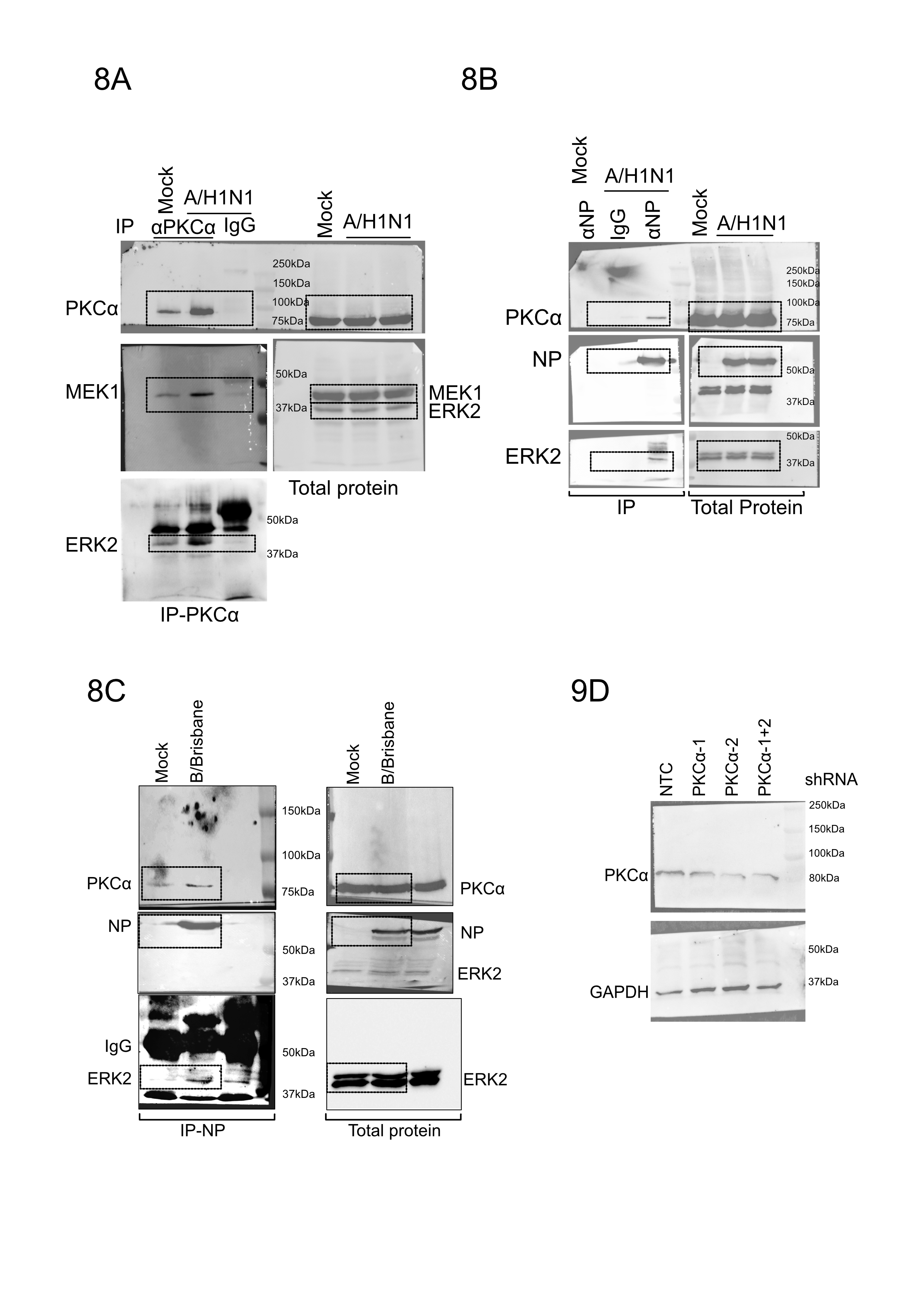


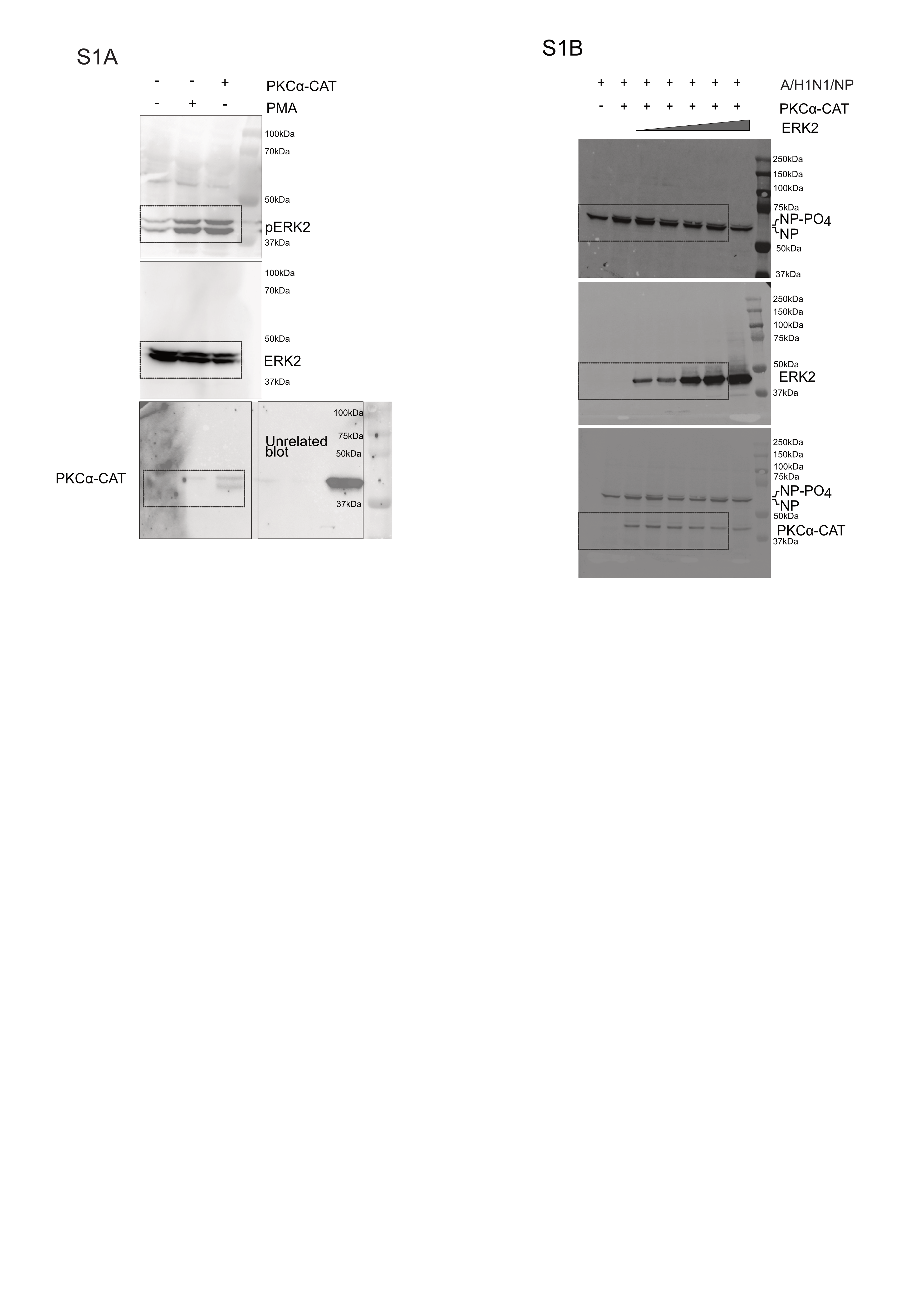


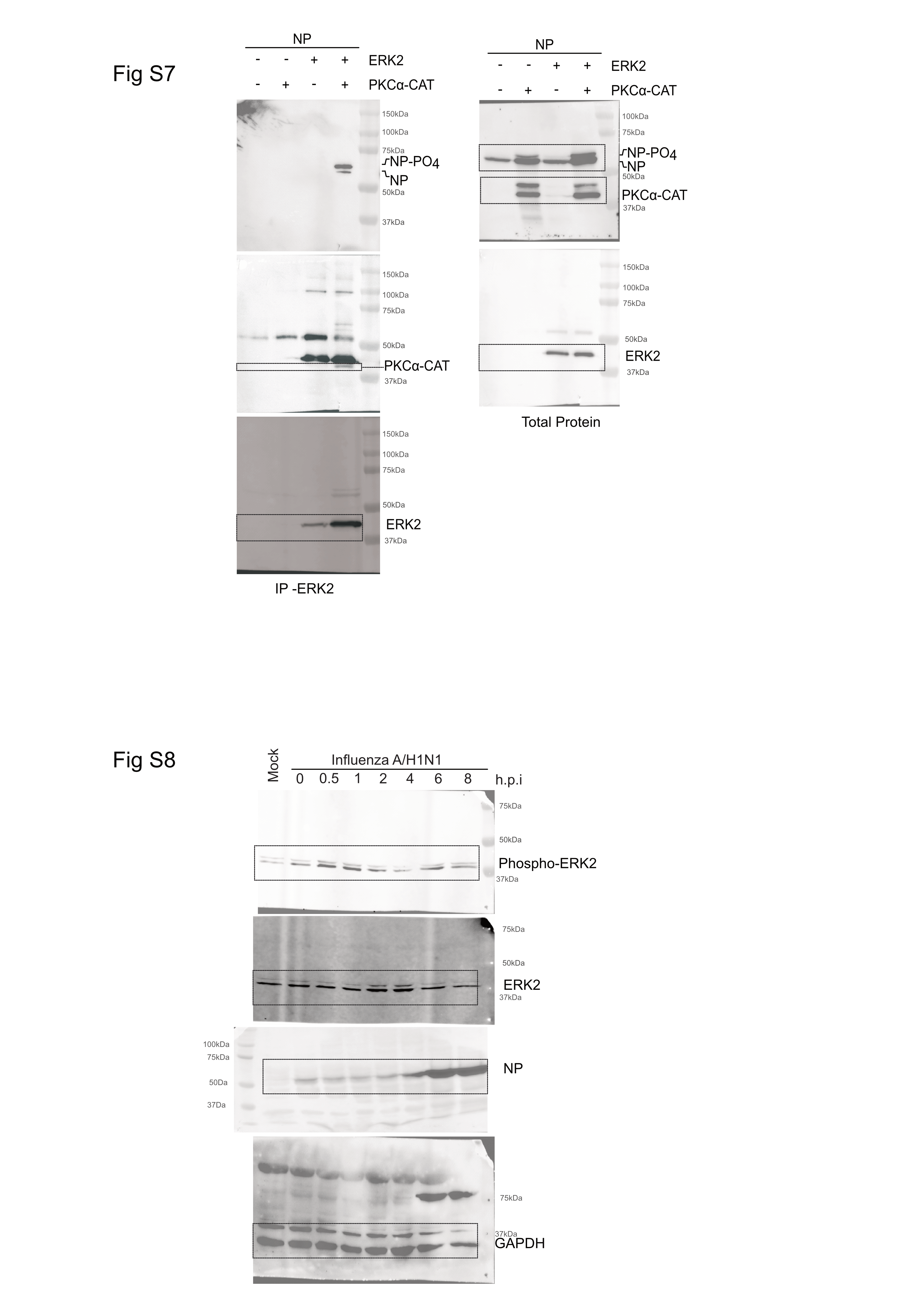

Supplement: S9 Table — (DOCX) [file ppat.1013841.s019.docx]
