# Supplementary material for: Host Protein Kinase C⍺: The novel Mitogen Activated Protein Kinase (MAPK) specific scaffold regulating nuclear export of influenza virus ribonucleoprotein complexes
Source: PLoS Pathog. 2025 Dec 31;21(12):e1013841. doi: 10.1371/journal.ppat.1013841 (PMC12788653; doi:10.1371/journal.ppat.1013841)

**S4 Table:** Ion series for the peptide: ATSPIVPSFDMSNEGSYFFGDNAEEYDN, T2-Phospho (79.96633Da) Charge: +3, Monoisotopic m/z: 1061.75223 Da


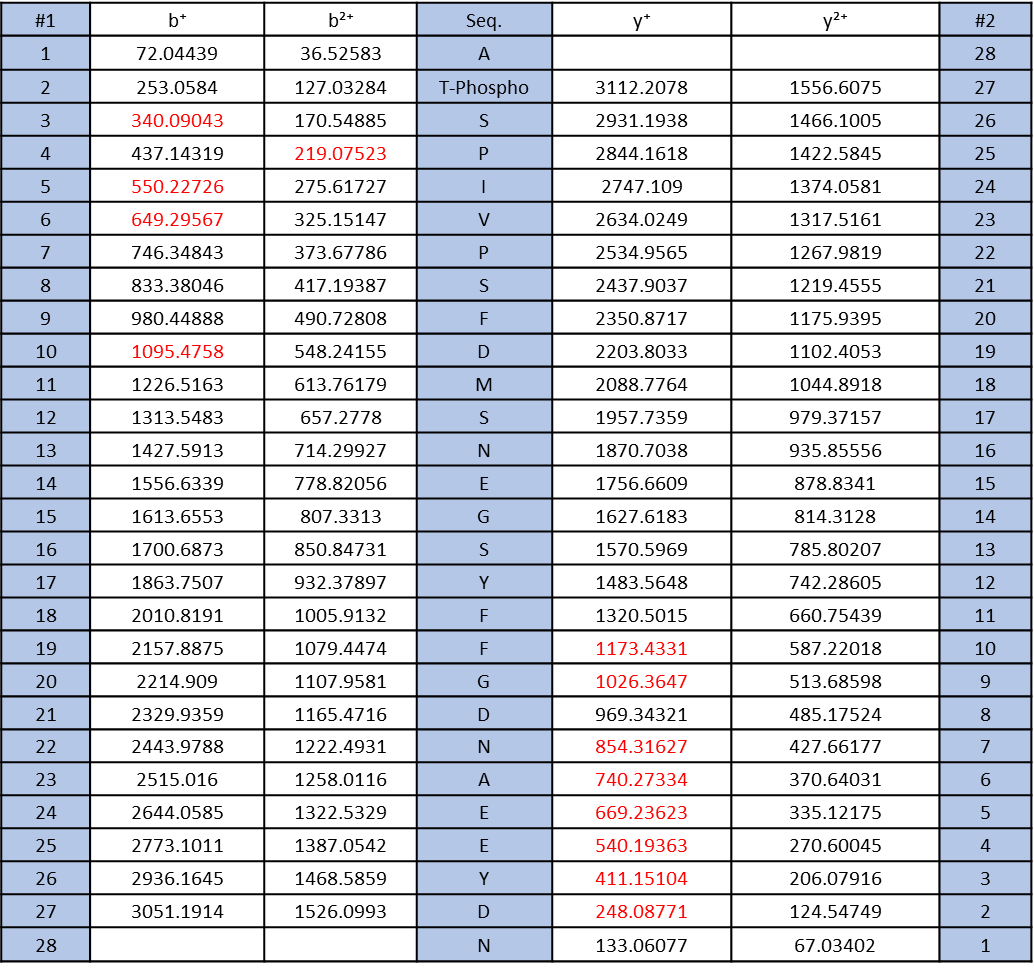

Supplement: S4 Table — (DOCX) [file ppat.1013841.s014.docx]
