# Supplementary material for: Host Protein Kinase C⍺: The novel Mitogen Activated Protein Kinase (MAPK) specific scaffold regulating nuclear export of influenza virus ribonucleoprotein complexes
Source: PLoS Pathog. 2025 Dec 31;21(12):e1013841. doi: 10.1371/journal.ppat.1013841 (PMC12788653; doi:10.1371/journal.ppat.1013841)
Supplement: S7 Table — (DOCX) [file ppat.1013841.s017.docx]

**S7 Table:** Percentage conservation of the ERK2 phosphorylation sites in different influenza virus NP proteins.

| **Residue No. (As per A/H1N1/WSN/1933)** | **Subtypes** | **% Conservation** |
| --- | --- | --- |
| S450 | Influenza A-H1N1 | 97.01652 |
|  | Influenza A-H5N1 | 100 |
|  | Influenza A-H7N9 | 100 |
|  | Influenza A-H3N2 | 9.725251 |
| S507 | Influenza B | 99.94 |
| S473 | A-H1N1 | 1.70394 |
|  | A-H5N1 | 0 |
|  | A-H7N9 | 0 |
|  | A-H3N2 | 0.65388 |
| T472 | A-H1N1 | 95 |
|  | A-H5N1 | 100 |
|  | A-H7N9 | 100 |
|  | A-H3N2 | 46.7 |
| T531 | Influenza B | 97.21 |
