# Supplementary material for: Host Protein Kinase C⍺: The novel Mitogen Activated Protein Kinase (MAPK) specific scaffold regulating nuclear export of influenza virus ribonucleoprotein complexes
Source: PLoS Pathog. 2025 Dec 31;21(12):e1013841. doi: 10.1371/journal.ppat.1013841 (PMC12788653; doi:10.1371/journal.ppat.1013841)
Supplement: S8 Table — (DOCX) [file ppat.1013841.s018.docx]

**S8 Table:** Coefficient of Co-localization from super resolution microscopy of PKC, MEK1 and ERK2 (in red) with NP (in green).

|  | Pearson's Coefficient | Manders' Coefficient | |
| --- | --- | --- | --- |
|  |  | M1  (fraction of Red overlapping Green) | M2  (fraction of Green overlapping Red) |
| ERK2 2HPI | 0.478 | 0.813 | 0.933 |
| ERK2 8HPI | 0.643 | 0.93 | 0.809 |
| MEK1 2HPI | 0.758 | 0.941 | 0.891 |
| MEK1 8HPI | 0.739 | 0.883 | 0.98 |
| PKC 2HPI | 0.54 | 0.92 | 0.9 |
| PKC 8HPI | 0.599 | 0.814 | 0.72 |
