# Supplementary material for: Host Protein Kinase C⍺: The novel Mitogen Activated Protein Kinase (MAPK) specific scaffold regulating nuclear export of influenza virus ribonucleoprotein complexes
Source: PLoS Pathog. 2025 Dec 31;21(12):e1013841. doi: 10.1371/journal.ppat.1013841 (PMC12788653; doi:10.1371/journal.ppat.1013841)
Supplement: Attachment — Submitted filename: Response to reviewers comments_20-8-25 .docx [file ppat.1013841.s021.docx]

Response to Reviewer’s comments:

We, the authors, would like express our sincere thanks to the Editor, Senior editor and Chief Editors for providing us the opportunity for revising and resubmitting the manuscript. We are also thankful to all three reviewers for praising our work in general and providing constructive comments, which helped us to improve the manuscript to a great extent. We have addressed all of the reviewer’s comments (below) by incorporating a new experimental data, extensively modifying and re-organising the main manuscript and including all of the source images in the supporting information as suggested. We are confident that with all these modifications, editors and reviewers would consider the manuscript suitable for publication in Plos Pathogens. The point by point response in as below.

Reviewers' Comments:

Reviewer's Responses to Questions

Part I - Summary
Please use this section to discuss strengths/weaknesses of study, novelty/significance, general execution and scholarship.

Reviewer #1: The manuscript by Das Jana et al provides relevant information about the interaction of host Kinase and the pathway involving ERK2, PKC alfa and other factors in the context of influenza, specifically through the interaction with NP. The work presented is scientifically sound and uses a combination of multiple techniques to delineate how ERK2 interacts with NP mechanistically, specific molecular signatures involved, how other host factors are or are not involved and the role of these interactions in the context of viral infections with influenza A but also influenza B. This is a well-written manuscript providing novel mechanistic data of the viral-host interactions of influenza that is relevant for the readers of PLOS Pathogens. Overall, I have outlined both significant and minor comments below that I would like the authors to address.

Major comments

The significant number of Western blots presented requires the inclusion of all whole membranes as supplementary material, which is also critical for the peer-review process.

Response: We have included the images of whole membranes of all the Western blots performed as supplementary material in Supporting information file.

Overall, the quality of the figures is poor. I made a significant effort to review this paper given the quality of the provided figures. I do not know if the issue relates to the original quality of the materials or the document generation for revision. Although I was able to capture most of the information, I cannot assess the IFI results presented in Figures 4, 7, and 9. A revised version should be submitted with higher-quality figures to facilitate a proper assessment of the results presented in the aforementioned figures.

Response: The composite file containing text and figures has low resolution images which seems to be more likely an issue with the software that generated the merged document. However, high resolution images of all of the figures could be downloaded from the link provided at the top right corner of the respective pages. However, we have resubmitted the high-resolution “Tiff” files of all the figures.

Minor comments

Line 327:…”Similar to ERK2, MEK1 but not MEK2 interacted with NP in a PKCα dependent manner”… Can the differences be explained by the total NP protein, clearly different between MEK1 and MEK2 experiments?

Response: We agree that the MEK2 sets has lower expression of NP than others. This can be explained by the fact that co-expression of ERK2 and PKCα-CAT with NP or co-expression of MEK1 and PKCα-CAT with NP always resulted not only in NP hyperphosphorylation but also in elevating the expression or stability of NP. This is why expression levels of NP remained significantly higher for these sets (compare Fig 5A, lane 2 versus lane 3 and Fig 5C, lane 2 versus lane 3). This data suggests that complex formation with PKCα, MEK1 and ERK2 not only phosphorylates NP but also stabilise the protein. As MEK2 cannot participate in this complex formation, therefore we can neither see NP hyperphosphorylation nor the boost in NP expression levels (Fig 5C, lane 4 and 5). So, the lower amount of NP in MEK2 sets might be due to the lack of extra stability of NP, as otherwise conferred by its interaction with the kinases like ERK2 or MEK1. A statement about this observation is included in the revised manuscript (line no:343-347).

The results presented about the role of ERK2 in the distribution of NP are impossible to assess due to the low quality of the pictures.
Can the limitation imposed by NP-Changes be overcome after passages of the virus?

Response: We have already addressed the issue related to the low

quality images that can be resolved upon downloading the high-resolution images. Also, the high-resolution “Tiff” files of all the figures have been resubmitted.

Influenza A virus NP can be phosphorylated by multiple host kinases including RSK1 (Schreiber et al., 2020) and ERK2 (our manuscript) both of which promote RNP nuclear export. These phosphorylation events might occur consecutively or independent of each other to aid RNP transport across nuclear membrane. While ERK2 phospho-mutant viruses might acquire adaptive changes during serial passaging, it might rather preferably use RSK1 mediated phosphorylation of NP to promote RNP nuclear export as an alternative strategy. Exploring these avenues are interesting but requires extensive investigation to elucidate how the activity of these kinases- ERK2 and RSK1, are coordinated to regulate RNP nuclear export, which is beyond the scope of the current study.

E339A role? Has it been addressed in the context of whole virus?

Response: Influenza A NP harboring E339A mutant lacks the critical E339-R416 slat-bridge interaction (Ye et al., 2006), which makes it completely defective in forming oligomers and supporting RNP assembly process. It has been shown by us as well as other groups in numerous reports that NP E339A mutant exists exclusively as monomer (Chenavas et al., 2013; Mondal et al., 2015, 2017) and hence recombinant virus harboring this mutation is not viable and cannot be rescued (Zejun et al., 2009).

..”Viral mRNA, cRNA and vRNA synthesis was significantly boosted for RNPs reconstituted with specific phosphomimetic variants of NPs (Fig 3I)”... It’s impossible to agree since comparisons with carrying A are not present.

Response: We have performed reporter -based polymerase activity assay with positive and negative sense reporter RNA templates either with WT or phosphonull alanine (A) or phosphomimetic glutamic acid (E) NP mutant proteins to reconstitute RNPs. Data presented in Figure 3G and 3H showed that all of the NP phosphonull (A) mutants supports RNA synthesis comparable to the WT while the NP mutants harboring more than one phosphomimetic substitutions (S450E/S473E and S450E/T472E/S473E) showed a consistent increase in RNP activity. Hence, these NP mutants (S450E/S473E and S450E/T472E/S473E) were specifically subjected to RNP reconstitution followed by primer extension analysis in Figure 3I to investigate if upregulation in RNA synthesis is specific to either viral mRNA transcription or viral genome replication step. Our data suggests that NP proteins harboring more than one phosphomimetic substitutions (S450E/S473E and S450E/T472E/S473E) show higher efficiency in supporting both the viral RNA synthesis events. As the phosphonull alanine mutants doesn’t show any change in reporter activity, hence they were excluded from the subsequent primer extension analysis.

Figure 1E shows a specific involvement of ERK2 in the phosphorylation of NP. However, the Western blots presented do not allow a firm conclusion about the expression of ERK2. Similar bands are observed when MEK1 is overexpressed, which should not be the case.

Response: In Figure 1E, we presented the in vitro kinasing experiment where FLAG epitope tagged ERK2, MEK1 or MEK2 proteins were individually overexpressed and purified from HEK293T cells (either stimulated with PMA or unstimulated) and subsequently used to phosphorylate recombinant purified NP in vitro. Top panel shows phosphor-image displaying the extent of NP phosphorylation in different sets; a robust NP phosphorylation was observed only for active ERK2 (stimulated with PMA) set but not for the unstimulated control set (lane 3 versus lane 4) or for the stimulated or unstimulated MEK1 or MEK2 sets (lanes 5- 8). The second panel presented Coomassie staining of the same gel as NP loading control. The lower most panel showed the western blot data of the whole cell extract collected before purification of each of the respective kinases to ensure that these kinases were expressed in the specific sets. **Since all the three kinases were FLAG epitope tagged, therefore we performed western blotting using anti FLAG antibody to show the expression of each of these kinases in specific sets of cells**. We have modified the figure legend to make experimental design more transparent. We hope this will address reviewer’s concern (line no. 1109-1111)

Results presented in figure 2H are hard to put into context due to the lack of NP wt (like in line 2 in figure 2G) and a condition without PKCδ. It is important to assess whether the detrimental effect observed in the presence of the mutant in figure 2G is reproducible for the experiments conducted in Fig 2H.

Response: Both in Figure 2G and 2H, we have purposefully used NP-E339A mutant as control instead of the WT NP. We reasoned that NP E339A being an obligatory monomeric protein should have maximum exposed phosphorylation sites. Some of these sites may remain inaccessible in case of the WT NP that remains as a mixture of monomer and oligomer. This notion is supported by the result presented in Figure 2A where NP E339A showed a 7.5 fold increase in ERK2 mediated NP phosphorylation compared to the WT NP. Similar results were also reported previously by Mondal et al (Mondal et al., 2017), where NP E339A was shown to be a superior substrate than NP WT for PKCδ mediated phosphorylation. This is why all the phosphonull mutations presented in Figure 2G and 2H were generated in the NP E339A plasmid backbone so that the effect of NP oligomerization and associated masking of the phosphosites could be nullified. Hence, NP E339A protein and not the WT NP was used as positive control for both of these experiments.

We agree with the reviewer’s second concern; hence we have repeated the experiment presented in figure 4H where an additional set without PKCδ has been included as negative control. Again, the results demonstrates that NP harboring phosphonull mutations at the ERK2 phosphorylation sites can be phosphorylated by PKCδ to the extent comparable to the NP E339A control. This data confirms that the phospho-sites reported in this manuscript can be phosphorylated by ERK2 only and not by PKCδ.

How is the level of replication between WT and the mutants for Figure 9? As presented, the results are misleading, as it was previously established that mutant replicates with a lower efficiency, which is not apparent in a graph normalized to 100%.

Response: We agree with the reviewer that the difference in replication levels of WT and mutant viruses are not evident in the relative percentage plots (Figure 9, E-G); these plots are included to highlight the change in replication of the WT and mutant viruses in control and PKCα knockdown cells. However, we have also included the absolute virus titre plots (Figure 9 H-J) where titre of WT and mutant viruses are plotted in Log10 scale without any normalization; these plots not only display the difference in replication of the WT and mutant viruses but also exhibit their differential sensitivity towards PKCα knockdown. These two sets of plots are two different representations of the same experimental result. We hope they together will address reviewer’s concern.

Please provide the primers used in the study.

Response: All primer sequences used in the study are incorporated in the Supporting information (Table S9).

The anti-RNP ab recognizes what exactly? Please provide more precise information. Is it an anti-PB2?

Response: Goat polyclonal antibody against influenza virus ribonucleoprotein (BEI Resources, no. NR-3133) was used to detect viral RNPs (mentioned in line no 602). It specifically detects influenza virus NP protein.

Line 248: The Authors mention a timeline for influenza infection that does not represent the experimental conditions used. The cited paper is based on human infections, not on in vitro systems utilizing highly permissive cells and laboratory-adapted strains.

Response: Thanks for raising this concern. Influenza virus A/ WSN/1933 (H1N1) completes a single cycle of replication by 8 hours. This has been changed in the manuscript and the reference has been updated (line no. 260). Also, keeping this timeline in mind, all of the infection experiments (Figure 4C, Figure 7, Figure 9A) were performed at early 2 hour and late 8 hours post infection. For Figure 8, the multi-protein complexes were monitored at 6 hours post infection.

Line 175 and 176: The text refers to figure 1H and not 1F

Response: Thank you for your suggestion, we have made the necessary changes (line no. 182,184).

Line 574: Spell out the strain A/PR/8/34

Response: Thank you for your suggestion, we have made the necessary changes (line no. 592).

Line 575-576: Was PMA added on top or the whole media was replaced with media + PMA?

Response: The whole media was replaced with DMEM supplemented with PMA for the respective sets (line 597-598).

Line 628: Provide more background information about pTMΔRNP plasmid and references.

Response: We have provided background information about pTMΔRNP plasmid and along with references (line no. 651-661).

Line 631: Provide composition of VGM.

Response: Thank you for your suggestion, we have made the necessary changes in the methods section as highlighted (line no. 662-663).

Line 653: Please modify Invitro to in vitro

Response: The word has been changed (line no: 685).

Reviewer #2: In this manuscript, the authors explore the mechanism of influenza A virus regulation by PKC alpha and the MAP kinase cascade, particularly NP export from the nucleus. The fact that PKC alpha and MAPK regulate replication was previously known, but the molecular players in the pathway had not yet been elucidated. Through in vitro and cell-based experiments, including both transfected and infected cells, the authors show that ERK2 is directly responsible for phosphorylating NP, but PKC alpha is required to activate ERK2 and also to act as a scaffold to bring ERK2, MEK1 and NP together. Moreover, they show that mutants that cannot be phosphorylated replicate less well, and that this is consistent with retention of NP in the nucleus at later times post infection, when NP should be moving to the cytoplasm (presumably as part of vRNPs). Similarly, they show that loss of PKC alpha activity reduces replication and NP export. The findings are significant because they provide a molecular pathway from PKC alpha to NP that explains regulation of influenza replication by this kinase and its downstream effectors. The experiments are generally strong and rigorous. I mostly have comments related to the writing and presentation.

Reviewer #3: In their manuscript Jana and colleagues elucidate the role of protein kinase C alpha in influenza virus RNP export. The paper is interesting but there are several points that need the authors’ attention.

Part II – Major Issues: Key Experiments Required for Acceptance
Please use this section to detail the key new experiments or modifications of existing experiments that should be absolutely required to validate study conclusions.

Generally, there should be no more than 3 such required experiments or major modifications for a "Major Revision" recommendation. If more than 3 experiments are necessary to validate the study conclusions, then you are encouraged to recommend "Reject".

Reviewer #1: The significant number of Western blots presented requires the inclusion of all whole membranes as supplementary material, which is also critical for the peer-review process.

Response: We have included all the images of whole membranes of Western blots in the supporting information.

Overall, the quality of the figures is poor. I made a significant effort to review this paper given the quality of the provided figures. I do not know if the issue relates to the original quality of the materials or the document generation for revision. Although I was able to capture most of the information, I cannot assess the IFI results presented in Figures 4, 7, and 9. A revised version should be submitted with higher-quality figures to facilitate a proper assessment of the results presented in the aforementioned figures.

Response: The composite file containing text and figures has low resolution images which seems to be more likely an issue with the software that generated the merged document. However, high resolution images of all of the figures could be downloaded from the link provided at the top right corner of the respective pages. However, we have resubmitted the high-resolution “Tiff” files of all the figures during the resubmission process.

Reviewer #2: 1. Lines 289-294 – this section is odd. What explains the change in localization of NP over time, in the absence of any stimulation? They have shown in Fig. 1 that NP phosphorylation in transfected cells requires PMA stimulation. Minimally, they should explain how they interpret this and why it is likely to be informative. Ideally, they should just show the data in infected cells, which is anyway more relevant. They may have to do additional experiments with the phosphomimetic mutants if they feel like this is an important point for their study.

Response:

**PMA stimulation (a generic activator of PKC),** has long been reported to influence NP nuclear export (Bui et al., 2002). The same can trigger NP hyperphosphorylation that can be visualised in SDS-PAGE through differential migration of the hyperphosphorylated and unphosphorylated (or minimally phosphorylated) NP bands (Mondal et al., 2017). In Fig. 1 we utilised PMA stimulation to show how activation of the PKC pathway influence the extent of NP hyperphosphorylation in ERK2 dependent manner. This helped us propose that PKC and MAPK activities act synergistically in order to phosphorylate NP.

**Monitoring NP localization in transfected cells (unstimulated):** Influenza virus NP harbors a bipartite nuclear localization signal (NLS 1-2) (Neumann et al., 1997; Weber et al., 1998; Wu et al., 2007) and three distinct nuclear export signals (NES 1-3) (Yu et al., 2012) which helps the protein to translocate in and out of the nucleus in absence of any other viral proteins. This is why NP expressed though transient transfection in cell gets gradually translocated into the nucleus during 10 to 24 hours post transfection and then exported out to the cytoplasm during 24 to 36 hours, even without any stimulation (see table below and current manuscript, Figure 4A). This dynamic subcellular localization of NP can be modulated by its phosphorylation. Previous literatures showed that phosphonull and phosphomimetic mutations at various phospho-sites of NP interfere with its localization in the cell (listed in the table below). Thus, monitoring NP subcellular localization in transfected cells is well accepted as an experimental tool to investigate the viral and host factors that are involved in regulating nuclear import and export of the influenza virus NP protein.

| **Sl. No.** | **Cell Line** | **Influenza virus strain** | **Nuclear localization of WT NP at hours post transfection** | **Cytoplasm localization of WT NP at hrs post transfection** | **Phosphosites in NP** | **Effect of phosphonull mutation upon subcellular localization** | **Effect of phosphomimetic mutation upon subcellular localization** | **Reference** |
| --- | --- | --- | --- | --- | --- | --- | --- | --- |
| 01 | 293T | Influenza A/WSN/1933 (H1N1) | 10 h p.t. | 24- 36 h p.t. | S9, Y10 | Phosphonull substitution results in nuclear retention at 10 h p.t | Phosphomimetic substitution results in early cytoplasmic translocation at 10 h p.t | (Zheng et al., 2015) |
| 02 | 293T | Influenza A/WSN/1933 (H1N1) | 12 h p.t. | 24- 36 h p.t. | Y296 | Nuclear retention caused by Y296E mutation at late time points | Accelerated  nuclear export caused by Y296F mutation at 36 h p.t. | (Zheng et al., 2015) |
| 03 | 293T | Influenza A/WSN/1933 (H1N1) | 12 h p.t. | 24- 36 h p.t. | Y78 | Y78F mutant exhibited nuclear localization at 12 h.p.t. and distributed in the cytoplasm at 24 and 36 h.p.t | Y78E mutant was located in nucleus at all time points | (Cui et al., 2019) |
| 04 | 293T | Influenza A/WSN/1933 (H1N1) | 10 h p.t. | 16, 24- 36 h p.t. | T188 | T188A mutants are displayed in cytoplasm at 36 h.p.t | T188E mutant NPs displayed obviously nuclear retention at 36 h.p.t | (Li et al., 2018) |

**Infection (stimulation) do influence NP localization:** We concur with reviewer’s concern that the stimulation/ activation of the kinases (PKCα or ERK2) might be more robust in case of infection rather than that of transfection. Possibly this is why the transfected NP proteins take around 24-36 hours to get exported out to the cytoplasm. Instead, NP/ RNP proteins get exported out to the cytoplasm as early as 4-6 hours post infection. Additionally, we saw higher cytoplasmic over nuclear abundance of WT NP in infected than in transfected cells but that was not the case for the phosphonull mutants (data presented below). This suggests that WT NP gets exported out of the nucleus more efficiently in infected cells than in transfected cells while the phophonull mutants are preferentially retained into the nucleus both in infected and transfected cells. Hence this data suggests that the defect in nuclear export of the NP phosphonull mutants are evident both in transfection and infection settings.

Though generating recombinant viruses harboring phosphomimetic residues in NP may provide additional supportive data, but could also raise biosafety concerns as NP harboring multiple phosphomimetic substitutions showed higher RNP activity and robust RNA synthesis than the WT and hence would be considered as gain of function for the virus (as shown in the Figure 3 G, H & I).

Figure legend: This figure is a representation of the data obtained from the nuclear cytoplasmic distribution of influenza A/WSN/1933 (H1N1) NP WT, S450A, S473A and S450A/ S473A proteins from the transfected (36 hpt) and infected (8hpi) data sets (Fig 4 B and C). As observed , the WT NP showed higher extent of nuclear export in infected set compared to the transfected set while the phosphonull mutants showed higher nuclear retention with no difference between infected and transfected sets.

Reviewer #3: Major points
1) The authors call PCKa a master regulator of the influenza virus life cycle. However, a virus lacking the appropriate phosphorylation sites grow as well as wild type. Since the virus seems to be fit without this whole interaction, the authors need to tone down their language.

Response: Our claim of PKCα being the master regulator for influenza virus life cycle is based upon the following experimental observations:

i) Data in Figure 3A and B showed that virus lacking both the ERK2 phosphosites (NP S450A/ S473A) is two log attenuated with respect to the (w.r.t) the WT virus, which unambiguously established the role of ERK2 mediated phosphorylation in supporting influenza virus replication.
ii) Subsequently data presented in Figure 5 & 6 showed that PKCα is indispensable for mediating the interaction between the upstream activating kinase MEK1, downstream effector kinase ERK2 and the substrate NP, thereby acting as a MAPK specific scaffold protein that facilitate ERK2 mediated NP phosphorylation.
iii) Data presented in Figure 9 A-C showed that overexpression of a dominant negative PKCα blocks RNP nuclear export and inhibits virus replication by more than two logs, thereby substantiating the importance of PKCα in supporting influenza virus replication. This is further supported by the fact that WT influenza viruses replicated poorly (one log lesser) in PKCα knockdown cells lines (Figure 9 E-G and H-J).
iv) Finally, we showed that the NP mutant viruses lacking ERK2 phosphosites, although replicated poorly with respect to the WT virus, but showed similar extent of replication in control and PKCα knockdown cells (Figure 9 E-G and H-J). This data established that PKCα activity is critical for ERK2 mediated NP phsphorylation, RNP nuclear export and virus replication.

We feel that all these data together cumulatively presented strong evidence towards the fact that PKCα is a key regulator of influenza virus replication.

**However, as per reviewer’s suggestion we have modified the title of the manuscript (line 1-3) to remove the word “master-regulator”.**

2) Many parts of the manuscript are written in a relatively sloppy way (see below), especially the methods section. This reflects bad on the conduct of the science itself too.

Response: We have extensively revised the writing as per reviewer’s suggestions and beyond.

3) It is unclear if the authors did their main experiments with A/WS/33 or A/WSN/33.

Response: The main infection experiments were performed Influenza A virus strains, A/ Wilson-Smith/1933 (H1N1) or A/WSN/1933 (H1N1) encoding PB2 with a C-terminal Flag (WSN-PB2-FLAG) (line 591-592).

4) The figures seem to be at a very low resolution.

Response:

The composite file containing text and figures has low resolution images which seems to be more likely an issue with the software that generated the merged document. However, high resolution images of all of the figures could be downloaded from the link provided at the top right corner of the respective pages. However, we have resubmitted the high-resolution “Tiff” files of all the figures during the resubmission process.

Part III – Minor Issues: Editorial and Data Presentation Modifications
Please use this section for editorial suggestions as well as relatively minor modifications of existing data that would enhance clarity.

Reviewer #1: Line 327:…”Similar to ERK2, MEK1 but not MEK2 interacted with NP in a PKCα dependent manner”… Can the differences be explained by the total NP protein, clearly different between MEK1 and MEK2 experiments?

The results presented about the role of ERK2 in the distribution of NP are impossible to assess due to the low quality of the pictures.
Can the limitation imposed by NP-Changes be overcome after passages of the virus?
E339A role? Has it been addressed in the context of whole virus?
..”Viral mRNA, cRNA and vRNA synthesis was significantly boosted for RNPs reconstituted with specific phosphomimetic variants of NPs (Fig 3I)”... It’s impossible to agree since comparisons with carrying A are not present.

Figure 1E shows a specific involvement of ERK2 in the phosphorylation of NP. However, the Western blots presented do not allow a firm conclusion about the expression of ERK2. Similar bands are observed when MEK1 is overexpressed, which should not be the case.
Results presented in figure 2H are hard to put into context due to the lack of NP wt (like in line 2 in figure 2G) and a condition without PKCδ. It is important to assess whether the detrimental effect observed in the presence of the mutant in figure 2G is reproducible for the experiments conducted in Fig 2H.
How is the level of replication between WT and the mutants for Figure 9? As presented, the results are misleading, as it was previously established that mutant replicates with a lower efficiency, which is not apparent in a graph normalized to 100%.
Please provide the primers used in the study.
The anti-RNP ab recognizes what exactly? Please provide more precise information. Is it an anti-PB2?
Line 248: The Authors mention a timeline for influenza infection that does not represent the experimental conditions used. The cited paper is based on human infections, not on in vitro systems utilizing highly permissive cells and laboratory-adapted strains.
Line 175 and 176: The text refers to figure 1H and not 1F
Line 574: Spell out the strain A/PR/8/34
Line 575-576: Was PMA added on top or the whole media was replaced with media + PMA?
Line 628: Provide more background information about pTMΔRNP plasmid and references.
Line 631: Provide composition of VGM.
Line 653: Please modify Invitro to in vitro

Response: This is repetition of the previous comments by Reviewer1.

Reviewer #2:

2. The author summary is too technical.

Response: We have significantly revised the “author summary” section (line 58-66).

3. In Fig. 1H, they do detect some phosphorylation in the cells that are lacking ERK2-AS. Is that cross-reactivity from the antibody? They should address it in the text.

Response: We do agree that both the empty vector control and the wildtype ERK2 catalysed marginal thiophosphate conjugation (phosphorylation using ATP-analogue) to NP, which has been detected using thiophospho-ester specific antibody. This can result from the high abundance of the ATP-analogue in cells during in-cell kinasing and hence utilisation of the same as an unfavourable substrate by endogenous ERK2 (for empty vector control) or overexpressed WT ERK2 to a minimal extent. As suggested, a statement describing the same has been included in the text (line no. 183-186).

4. Line 205 – I assume they mean NP S473?

Response: Yes, the line has been modified accordingly (line 214).

5. Fig. 2E – in the diagram, should it not be T472?

Response: The figure has been modified accordingly.

6. In Fig. 2G, the top panel (pNP) has some cropped lanes but the others do not seem to. First of all, the authors should make it more obvious if they cropped lanes (for example use a dashed line). Second, are these from the same experiment? Why were lane not cropped for the other proteins?

Response: The Figure 2G, lane 6 has a white line passing though the left edge of the phospho-NP band. This is not a cropped image, rather it is an impression of the phosphor imager screen which unfortunately has been incorporated in the image. The full uncropped image of the same has been included in the Supporting information which shows the same line across the gel. Notably the line could be seen passing through the lane 6, which should not be the case for a cropped image.

7. In the legend for Fig. 2D,E,F, they should specify the full name of the strains from which they got the NP sequences, not just the subtype.

Response: Changes have been incorporated accordingly in the figure legend (line 1123-1132).

8. Fig. 2 legend, line 1056 – they should specify that the plotted measurements are normalized to ERK2 phosphorylation of WT NP (alternatively, they can specify that in the axis label of Fig. 2A)

Response: A statement describing the same has been incorporated in the Figure 2 legend (line no. 1119).

9. Line 232 – I am not sure I agree with the statement that the mutants have lower replication kinetics – they initially look the same, but never reach the same level. It is unclear if they ever would.

Response: A more clear and descriptive statement (mentioned below) has been incorporated at the line no. 240-245.

“the single mutants showed replication comparable to the WT virus at early time points (8-24 hours) but one log attenuation by 48-72 hours post infection (hpi). Viruses harboring the double mutant NP-S450A/S473A showed attenuation as early as 16 hours and the most drastic two log attenuation by 48 hpi onwards.”

10. Line 244 – this sentence should be rephrased because it makes it sound like there is less of a reduction in vRNA synthesis at 48 hpi, as grammatically the 5% refers to reduction. What they mean is a 95% reduction.

Response: The line has been modified accordingly (line no: 254-256).

11. Lines 258-260 – It is not clear what they think causes the positive regulation of RNA synthesis with the phosphomimetic mutants.

Response: The precise mechanism by which NP molecules harboring multiple phosphomimetic mutations showed higher extent of RNA synthesis warrants extensive investigation. However, considering that NP can only impact RNA synthesis through its ability to support RNP assembly, primarily it can be inferred that these phosphomimetic NP proteins may serve as a superior substrate for the assembly of functional RNPs as mentioned in the line no. 276-277.

12. Fig, 3 – the graphs showing time points of replication/viral genome (panels B, D, and F) should be plotted in an actual linear scale – right now the x-axis is a categorical variable, with equal distances representing 5 hrs to 24 hrs.

Response: As suggested, necessary changes are being made in the Figure 3.

13. Fig. 3 legend – Would be good if they specified the multiplicity of infection for assays in panels C to F.

Response: Necessary changes are incorporated in the methodology section as well as in the figure legend (line no: 859 and 1142-1143).

14. Lines 302-303 – they should clarify how these data show that the events are independent of RSK1, I am not clear why.

Response: We concur with the reviewer’s concern. It is not possible to make any conclusive statement about RSK1’s involvement from the data presented in the manuscript. Hence, this specific part of the sentence has been deleted (line no. 314).

15. Fig. 4C, right – it would be more informative to show significance against WT, instead of between the mutants

Response: We thank the reviewer for the suggestion. The graph in Fig 4C has been revised accordingly.

16. Fig. 4, 7, 9B legends – they should specify how many biological replicates of the microscopy experiments they did, because the reference to fields makes it sound like it was 1, which is not sufficient.

Response: We appreciate the reviewer for raising this concern. Each experiment was repeated at least 3 times. One such representative image was incorporated in the manuscript. 50 cells from 5 different fields were analysed for each independent experiment. This is now explicitly stated in the methodology section as well as in the figure legends of Fig 4,7 and 9. (line no: 754,1161, 1196 and 1230-1231).

17. Line 307 – the sentence and section start with “To this end,” but it’s not clear what end they are talking about.

Response: We have rephrased this sentence in the manuscript (line no: 318-319).

18. Line 350 – Should be “it failed to…”

Response: The line has been modified accordingly (line no: 367).

19. In the diagrams in Fig. 6, the inhibitory arrows from NP/ERK2 to inactive PKC alpha are confusing because they suggest that NP/ERK2 are preventing something, whereas it is actually the lack of active PKC alpha that is preventing the interaction. They should find a different way to represent the missing interaction.

Response: We have replaced the inhibitory arrow with a red cross in Fig. 6 A, C for better clarity.

20. Throughout the manuscript, they should have a space between numbers and units (48 hpi, not 48hpi for example). Currently they have it some of the time but not all of the time.

Response: We have made changes throughout the manuscript.

21. Line 534 – they should rephrase “ERK downstream RSK1 mediated NP phosphorylation” because it is hard to follow.

Response: The line has been rephrased accordingly for better clarity (line no: 552-553).

22. Line 651, 653 – should be in vitro instead of invitro.

Response: The line has been modified accordingly (line no: 683, 685).

23. Line 712 – how did they define the nucleus vs. cytoplasmic compartments?

Response: We measured the nuclear intensity of NP by measuring the NP signal intensity overlapping with DAPI, a nuclear marker. We didn’t measure cytoplasmic intensity separately, instead we measured the total NP intensity throughout the cell. Thereafter, we calculated the ratio of nuclear NP intensity upon total NP intensity for each individual cell and plotted on the Y axis of the graph. The increase in the ratio indicates higher nuclear distribution and vice versa.

Reviewer #3: Minor points

1) Lines 33, 85, 108, 566, 567, 582 : There is no need to start words with capitalized letters mid-sentence.

Response: Thank you for your suggestion, we have modified the words accordingly (line no. 34, 87, 110, 585, 586, 604).

2) Line 34: Please define Raf, MEK, ERK and MAPK.

Response: Thank you for your suggestion, we have defined the words accordingly (line no. 35-37).

3) Line 48: ‘a’, not ‘the’ – also see comment above about key regulator.

Response: The word has been replaced (line no: 50). Please see response to that specific comment.

4) Lines 59, 145-147, 191-194 : Please rephrase these sentences.

Response: These sentences are rephrased (line no. 61,148-151, 299-202).

5) Line 71: Please tone that down. Current antivirals work well against almost all strains. Vaccines work too, with caveats.

Response: Thank you for your suggestion, we have changed the statement (line no: 71-73).

6) Line 77: ‘gets’, not ‘get’

Response: “Eight negative sense viral genomic RNA segments, individually gets enwrapped with nucleoprotein (NP) oligomers”. Didn’t find ‘get’ (line no: 79).

7) Line 89: ‘phosphorylations’

Response: The word has been corrected (line no: 91).

8) Throughout the manuscript: Please define abbreviations when first used. This includes: Raf, MEK, ERK, HA, HEK, SDS-PAGE, ATP, MDCK, hpi, FBS, DMSO, UTRs, BNP

Response: Thank you for your suggestion, we have made necessary changes throughout the manuscript accordingly.

9) Line 97: ‘the MAPK pathway’

Response: The word has been incorporated (line no: 99).

10) Line 98: ‘of the virus life cycle’

Response: The word has been incorporated (line no: 100).

11) Line 114: ‘facilitates’

Response: The word has been replaced (line no: 116).

12) Line 121: ‘modulates’

Response: The word has been replaced (line no: 123).

13) Line 123: ‘phosphorylates’

Response: The word has been replaced (line no: 125).

14) Lines 125, 142, 157, 158, 160, 322, 381, 397, 401, 481, 571: Many different versions of the strain name A/WSN/1933 are used throughout the manuscript. Please stick to ‘A/WSN/1933 (H1N1)’.

Response: Thank you for your suggestion, we have made necessary changes throughout the manuscript.

15) Line 153: ‘an in vitro’

Response: The word has been incorporated (line no: 157).

16) Line 159: ‘an extend’ not ‘the extend’

Response: The word has been replaced (line no: 163).

17) Line 163: ‘the virus life cycle’

Response: The word has been incorporated (line no: 168).


18) Line 169: ‘MOI’, not ‘MO1’

Response: The word has been replaced (line no: 174).


19) Line 169: ‘with PMA’

Response: The word has been replaced (line no: 175).


20) Line 171: ‘with thio-phosphate’

Response: Changed (line no-178)

21) Line 198: ‘does not’, not ‘doesn’t’

Response: The word has been replaced (line no: 206).


22) Line 238: ‘of the influenza virus’

Response: The word has been incorporated (line no: 249).


23) Line 240: Remove ‘of’

Response: The word has been removed (line no: 251).


24) Line 247: ‘showed a trend’

Response: The word has been incorporated (line no: 258).


25) Line 251: ‘of the virus’

Response: The word has been incorporated (line no: 263).


26) Line 257: ‘to a defect’

Response: The word has been incorporated (line no: 268).


27) Line 274: ‘showing a minor’

Response: The word has been incorporated (line no: 288).


28) Line 286: ‘phospho’ is misspelled.

Response: The word has been corrected (line no: 298).


29) Line 289: ‘a potential role’

Response: The word has been incorporated (line no: 301).


30) Line 291: ‘render’

Response: The word has been corrected (line no: 306).


31) Line 294: ‘showed an intermediate’

Response: The word has been incorporated (line no: 303).


32) Line 322: Please use proper strain names.

Response: The changes have been incorporated as suggested (line no: 334-335).

33) Line 331: ‘of the PB2’

Response: The word has been incorporated (line no: 348).


34) Line 339: ‘mediating’

Response: Changed (line no. 356).

35) Line 343: Remove ‘the after ‘utilised’.

Response: The word has been removed (line no: 360).


36) Line 348: ‘mutations’

Response: The word has been incorporated (line no: 365).


37) Line 389: ‘the majority’

Response: The word has been incorporated (line no: 406).


38) Line 398: ‘infected set’?

Response: We have rephrased the sentence for better understanding (line no: 415-416).


39) Line 400: Remove the comma after ‘if’.

Response: removed (line no. 417)

40) Line 428: Remove ‘of’ after ‘times’.

Response: The word has been removed (line no: 446).

41) Line 429: Please rephrase this sentence.

Response: Rephrased (line no. 446- 448).

42) Line 475: ‘flight’ is not the right word here.

Response: The word has been replaced with export (line no: 493).


43) Line 478: ‘to the cytoplasm’

Response: The word has been incorporated (line no: 493).


44) Line 486: ‘supporting the virus’

Response: The word has been incorporated (line no: 505).


45) Line 499: Remove the ‘if’ after ‘that’.

Response: “It is noteworthy that, both ERK2 and its downstream kinase RSK1 phosphorylates distinct residues in NP to promote vRNP nuclear export.” There is no “if” after “that” (line no 518).

46) Line 501: ‘of the MAPK cascade’

Response: The words have been incorporated (line no: 520).


47) Line 502: ‘of the infection’

Response: The word has been incorporated (line no: 521).

48) Line 535: Should be ‘promotes’ and ‘the vRNP’

Response: The words have been incorporated (line no: 550-551S).

49) Line 548: ‘the virion’

Response: The word has been incorporated (line no: 572).

50) Line 558: ‘detailed’

Response: The word has been incorporated (line no: 577).


51) Line 569: What is the concentration of antimycotic (and which one was used), penicillin and streptomycin?

Response: The concentrations have been incorporated as suggested (line no: 588-589).


52) Line 572: Is ‘FLAG tag’ meant?

Response: The sentence has been rephrased for clarity (line no. 591-593).

53) Line 572: There is a space missing.

Response: The space has been incorporated after the word (line no: 599).


54) Line 585: ‘virus’

Response: The word has been corrected (line no: 613).


55) Line 586: There is a space missing.

Response: The space has been incorporated after the word (line no: 608).


56) Line 590: ‘the full-length’

Response: The word has been incorporated (line no: 612).


57) This reviewer gave up pointing out issues after line 590 – but it did not get any better.

Response: We continued to make changes as per our best possible effort. Hopefully with all the changes made, the current version of the manuscript would be suitable for acceptance. We sincerely thank the reviewer for his/her time and effort to make the manuscript significantly better than its original version.

**References:**

Bui, M., Myers, J. E., & Whittaker, G. R. (2002). *Nucleo-cytoplasmic localization of influenza virus nucleoprotein depends on cell density and phosphorylation*. *84*, 37–44.

Chenavas, S., Estrozi, L. F., Slama-Schwok, A., Delmas, B., Di Primo, C., Baudin, F., Li, X., Crépin, T., & Ruigrok, R. W. H. (2013). Monomeric nucleoprotein of influenza A virus. *PLoS Pathogens*, *9*(3), e1003275. https://doi.org/10.1371/journal.ppat.1003275

Cui, L., Zheng, W., Li, M., Bai, X., Yang, W., Li, J., Fan, W., Gao, G. F., Sun, L., & Liu, W. (2019). Phosphorylation Status of Tyrosine 78 Residue Regulates the Nuclear Export and Ubiquitination of Influenza A Virus Nucleoprotein. *Frontiers in Microbiology*, *10*(August). https://doi.org/10.3389/fmicb.2019.01816

Li, Y., Sun, L., Zheng, W., Madina·Mahesutihan, Li, J., Bi, Y., Wang, H., Liu, W., & Luo, T. R. (2018). Phosphorylation and dephosphorylation of threonine 188 in nucleoprotein is crucial for the replication of influenza A virus. *Virology*, *520*, 30–38. https://doi.org/https://doi.org/10.1016/j.virol.2018.05.002

Mondal, A., Dawson, A. R., Potts, G. K., Freiberger, E. C., Baker, S. F., Moser, L. A., Bernard, K. A., Coon, J. J., & Mehle, A. (2017). Influenza virus recruits host protein kinase C to control assembly and activity of its replication machinery. *ELife*, *6*, 1–23. https://doi.org/10.7554/eLife.26910.001

Mondal, A., Potts, G. K., Dawson, A. R., Coon, J. J., & Mehle, A. (2015). Phosphorylation at the Homotypic Interface Regulates Nucleoprotein Oligomerization and Assembly of the Influenza Virus Replication Machinery. *PLoS Pathogens*, *11*(4), 1–24. https://doi.org/10.1371/journal.ppat.1004826

Neumann, G., Castrucci, M. R., & Kawaoka, Y. (1997). Nuclear import and export of influenza virus nucleoprotein. *Journal of Virology*, *71*(12), 9690–9700. https://doi.org/10.1128/jvi.71.12.9690-9700.1997

Schreiber, A., Boff, L., Anhlan, D., Krischuns, T., Brunotte, L., Schuberth, C., Wedlich-Söldner, R., Drexler, H., & Ludwig, S. (2020). Dissecting the mechanism of signaling-triggered nuclear export of newly synthesized influenza virus ribonucleoprotein complexes. *Proceedings of the National Academy of Sciences of the United States of America*, *117*(28), 16557–16566. https://doi.org/10.1073/pnas.2002828117

Weber, F., Kochs, G., Gruber, S., & Haller, O. (1998). A classical bipartite nuclear localization signal on Thogoto and influenza A virus nucleoproteins. *Virology*, *250*(1), 9–18. https://doi.org/10.1006/viro.1998.9329

Wu, W. W. H., Sun, Y. H. B., & Panté, N. (2007). Nuclear import of influenza A viral ribonucleoprotein complexes is mediated by two nuclear localization sequences on viral nucleoprotein. *Virology Journal*, *4*, 1–12. https://doi.org/10.1186/1743-422X-4-49

Ye, Q., Krug, R. M., & Tao, Y. J. (2006). The mechanism by which influenza A virus nucleoprotein forms oligomers and binds RNA. *Nature*, *444*(7122), 1078–1082. https://doi.org/10.1038/nature05379

Yu, M., Liu, X., Cao, S., Zhao, Z., Zhang, K., Xie, Q., Chen, C., Gao, S., Bi, Y., Sun, L., Ye, X., Gao, G. F., & Liu, W. (2012). Identification and characterization of three novel nuclear export signals in the influenza A virus nucleoprotein. *Journal of Virology*, *86*(9), 4970–4980. https://doi.org/10.1128/JVI.06159-11

Zejun, L., Tokiko, W., Masato, H., Shinji, W., Asuka, N., Makoto, O., Satoshi, K., Masayuki, S., Shinya, Y., Gabriele, N., & Yoshihiro, K. (2009). Mutational Analysis of Conserved Amino Acids in the Influenza A Virus Nucleoprotein. *Journal of Virology*, *83*(9), 4153–4162. https://doi.org/10.1128/jvi.02642-08

Zheng, W., Li, J., Wang, S., Cao, S., Jiang, J., Chen, C., Ding, C., Qin, C., Ye, X., Gao, G. F., & Liu, W. (2015). Phosphorylation controls the nuclear-cytoplasmic shuttling of influenza A virus nucleoprotein. *Journal of Virology*, *89*(11), 5822–5834. https://doi.org/10.1128/JVI.00015-15

Bui, M., Myers, J. E., & Whittaker, G. R. (2002). *Nucleo-cytoplasmic localization of influenza virus nucleoprotein depends on cell density and phosphorylation*. *84*, 37–44.

Chenavas, S., Estrozi, L. F., Slama-Schwok, A., Delmas, B., Di Primo, C., Baudin, F., Li, X., Crépin, T., & Ruigrok, R. W. H. (2013). Monomeric nucleoprotein of influenza A virus. *PLoS Pathogens*, *9*(3), e1003275. https://doi.org/10.1371/journal.ppat.1003275

Cui, L., Zheng, W., Li, M., Bai, X., Yang, W., Li, J., Fan, W., Gao, G. F., Sun, L., & Liu, W. (2019). Phosphorylation Status of Tyrosine 78 Residue Regulates the Nuclear Export and Ubiquitination of Influenza A Virus Nucleoprotein. *Frontiers in Microbiology*, *10*(August). https://doi.org/10.3389/fmicb.2019.01816

Li, Y., Sun, L., Zheng, W., Madina·Mahesutihan, Li, J., Bi, Y., Wang, H., Liu, W., & Luo, T. R. (2018). Phosphorylation and dephosphorylation of threonine 188 in nucleoprotein is crucial for the replication of influenza A virus. *Virology*, *520*, 30–38. https://doi.org/https://doi.org/10.1016/j.virol.2018.05.002

Mondal, A., Dawson, A. R., Potts, G. K., Freiberger, E. C., Baker, S. F., Moser, L. A., Bernard, K. A., Coon, J. J., & Mehle, A. (2017). Influenza virus recruits host protein kinase C to control assembly and activity of its replication machinery. *ELife*, *6*, 1–23. https://doi.org/10.7554/eLife.26910.001

Mondal, A., Potts, G. K., Dawson, A. R., Coon, J. J., & Mehle, A. (2015). Phosphorylation at the Homotypic Interface Regulates Nucleoprotein Oligomerization and Assembly of the Influenza Virus Replication Machinery. *PLoS Pathogens*, *11*(4), 1–24. https://doi.org/10.1371/journal.ppat.1004826

Neumann, G., Castrucci, M. R., & Kawaoka, Y. (1997). Nuclear import and export of influenza virus nucleoprotein. *Journal of Virology*, *71*(12), 9690–9700. https://doi.org/10.1128/jvi.71.12.9690-9700.1997

Schreiber, A., Boff, L., Anhlan, D., Krischuns, T., Brunotte, L., Schuberth, C., Wedlich-Söldner, R., Drexler, H., & Ludwig, S. (2020). Dissecting the mechanism of signaling-triggered nuclear export of newly synthesized influenza virus ribonucleoprotein complexes. *Proceedings of the National Academy of Sciences of the United States of America*, *117*(28), 16557–16566. https://doi.org/10.1073/pnas.2002828117

Weber, F., Kochs, G., Gruber, S., & Haller, O. (1998). A classical bipartite nuclear localization signal on Thogoto and influenza A virus nucleoproteins. *Virology*, *250*(1), 9–18. https://doi.org/10.1006/viro.1998.9329

Wu, W. W. H., Sun, Y. H. B., & Panté, N. (2007). Nuclear import of influenza A viral ribonucleoprotein complexes is mediated by two nuclear localization sequences on viral nucleoprotein. *Virology Journal*, *4*, 1–12. https://doi.org/10.1186/1743-422X-4-49

Ye, Q., Krug, R. M., & Tao, Y. J. (2006). The mechanism by which influenza A virus nucleoprotein forms oligomers and binds RNA. *Nature*, *444*(7122), 1078–1082. https://doi.org/10.1038/nature05379

Yu, M., Liu, X., Cao, S., Zhao, Z., Zhang, K., Xie, Q., Chen, C., Gao, S., Bi, Y., Sun, L., Ye, X., Gao, G. F., & Liu, W. (2012). Identification and characterization of three novel nuclear export signals in the influenza A virus nucleoprotein. *Journal of Virology*, *86*(9), 4970–4980. https://doi.org/10.1128/JVI.06159-11

Zejun, L., Tokiko, W., Masato, H., Shinji, W., Asuka, N., Makoto, O., Satoshi, K., Masayuki, S., Shinya, Y., Gabriele, N., & Yoshihiro, K. (2009). Mutational Analysis of Conserved Amino Acids in the Influenza A Virus Nucleoprotein. *Journal of Virology*, *83*(9), 4153–4162. https://doi.org/10.1128/jvi.02642-08

Zheng, W., Li, J., Wang, S., Cao, S., Jiang, J., Chen, C., Ding, C., Qin, C., Ye, X., Gao, G. F., & Liu, W. (2015). Phosphorylation controls the nuclear-cytoplasmic shuttling of influenza A virus nucleoprotein. *Journal of Virology*, *89*(11), 5822–5834. https://doi.org/10.1128/JVI.00015-15

Bui, M., Myers, J. E., & Whittaker, G. R. (2002). *Nucleo-cytoplasmic localization of influenza virus nucleoprotein depends on cell density and phosphorylation*. *84*, 37–44.

Chenavas, S., Estrozi, L. F., Slama-Schwok, A., Delmas, B., Di Primo, C., Baudin, F., Li, X., Crépin, T., & Ruigrok, R. W. H. (2013). Monomeric nucleoprotein of influenza A virus. *PLoS Pathogens*, *9*(3), e1003275. https://doi.org/10.1371/journal.ppat.1003275

Cui, L., Zheng, W., Li, M., Bai, X., Yang, W., Li, J., Fan, W., Gao, G. F., Sun, L., & Liu, W. (2019). Phosphorylation Status of Tyrosine 78 Residue Regulates the Nuclear Export and Ubiquitination of Influenza A Virus Nucleoprotein. *Frontiers in Microbiology*, *10*(August). https://doi.org/10.3389/fmicb.2019.01816

Li, Y., Sun, L., Zheng, W., Madina·Mahesutihan, Li, J., Bi, Y., Wang, H., Liu, W., & Luo, T. R. (2018). Phosphorylation and dephosphorylation of threonine 188 in nucleoprotein is crucial for the replication of influenza A virus. *Virology*, *520*, 30–38. https://doi.org/https://doi.org/10.1016/j.virol.2018.05.002

Mondal, A., Dawson, A. R., Potts, G. K., Freiberger, E. C., Baker, S. F., Moser, L. A., Bernard, K. A., Coon, J. J., & Mehle, A. (2017). Influenza virus recruits host protein kinase C to control assembly and activity of its replication machinery. *ELife*, *6*, 1–23. https://doi.org/10.7554/eLife.26910.001

Mondal, A., Potts, G. K., Dawson, A. R., Coon, J. J., & Mehle, A. (2015). Phosphorylation at the Homotypic Interface Regulates Nucleoprotein Oligomerization and Assembly of the Influenza Virus Replication Machinery. *PLoS Pathogens*, *11*(4), 1–24. https://doi.org/10.1371/journal.ppat.1004826

Neumann, G., Castrucci, M. R., & Kawaoka, Y. (1997). Nuclear import and export of influenza virus nucleoprotein. *Journal of Virology*, *71*(12), 9690–9700. https://doi.org/10.1128/jvi.71.12.9690-9700.1997

Schreiber, A., Boff, L., Anhlan, D., Krischuns, T., Brunotte, L., Schuberth, C., Wedlich-Söldner, R., Drexler, H., & Ludwig, S. (2020). Dissecting the mechanism of signaling-triggered nuclear export of newly synthesized influenza virus ribonucleoprotein complexes. *Proceedings of the National Academy of Sciences of the United States of America*, *117*(28), 16557–16566. https://doi.org/10.1073/pnas.2002828117

Weber, F., Kochs, G., Gruber, S., & Haller, O. (1998). A classical bipartite nuclear localization signal on Thogoto and influenza A virus nucleoproteins. *Virology*, *250*(1), 9–18. https://doi.org/10.1006/viro.1998.9329

Wu, W. W. H., Sun, Y. H. B., & Panté, N. (2007). Nuclear import of influenza A viral ribonucleoprotein complexes is mediated by two nuclear localization sequences on viral nucleoprotein. *Virology Journal*, *4*, 1–12. https://doi.org/10.1186/1743-422X-4-49

Ye, Q., Krug, R. M., & Tao, Y. J. (2006). The mechanism by which influenza A virus nucleoprotein forms oligomers and binds RNA. *Nature*, *444*(7122), 1078–1082. https://doi.org/10.1038/nature05379

Yu, M., Liu, X., Cao, S., Zhao, Z., Zhang, K., Xie, Q., Chen, C., Gao, S., Bi, Y., Sun, L., Ye, X., Gao, G. F., & Liu, W. (2012). Identification and characterization of three novel nuclear export signals in the influenza A virus nucleoprotein. *Journal of Virology*, *86*(9), 4970–4980. https://doi.org/10.1128/JVI.06159-11

Zejun, L., Tokiko, W., Masato, H., Shinji, W., Asuka, N., Makoto, O., Satoshi, K., Masayuki, S., Shinya, Y., Gabriele, N., & Yoshihiro, K. (2009). Mutational Analysis of Conserved Amino Acids in the Influenza A Virus Nucleoprotein. *Journal of Virology*, *83*(9), 4153–4162. https://doi.org/10.1128/jvi.02642-08

Zheng, W., Li, J., Wang, S., Cao, S., Jiang, J., Chen, C., Ding, C., Qin, C., Ye, X., Gao, G. F., & Liu, W. (2015). Phosphorylation controls the nuclear-cytoplasmic shuttling of influenza A virus nucleoprotein. *Journal of Virology*, *89*(11), 5822–5834. https://doi.org/10.1128/JVI.00015-15
