# Supplementary material for: Host Protein Kinase C⍺: The novel Mitogen Activated Protein Kinase (MAPK) specific scaffold regulating nuclear export of influenza virus ribonucleoprotein complexes
Source: PLoS Pathog. 2025 Dec 31;21(12):e1013841. doi: 10.1371/journal.ppat.1013841 (PMC12788653; doi:10.1371/journal.ppat.1013841)
Supplement: Attachment — Submitted filename: Response to reviewers_28-11-2025.docx [file ppat.1013841.s022.docx]

**Reviewers' Comments:**

Reviewer's Responses to Questions

**Part I - Summary**
Please use this section to discuss strengths/weaknesses of study, novelty/significance, general execution and scholarship.

Reviewer #4: In this revised manuscript, the authors have adequately addressed the previous reviewers' relatively minor concerns through revisions to the text and new experimental data. Overall, their results support their conclusions and the data are presented in an easily interpretable manner. The model schematics in multiple figures help the reader interpret the experimental design and results. Congrats on this interesting study.

Reviewer #5: In this article, novel influenza virus-host interactions are described. Specifically, the authors show that the ERK2 kinase phosphorylates the influenza NP protein, and they identify the NP residues being phosphorylated by ERK2. In addition, they show that the NP phosphorylation is essential for vRNP export from the nucleus to the cytoplasm, and for virus propagation at late times post-infection. Furthermore, they show that PKC⍺ activates the MAPK cascade (leading to ERK2 activation), and PKC⍺ participates in stable interactions with the upstream kinase, MEK1, with ERK2, and with the influenza NP, thereby forming a multiprotein complex that regulates ERK2 activation, and NP phosphorylation. The results will be interesting for virologists and cellular biologists in general. However, there are some points which should be considered before publication.

Reviewer #6: Using an in vitro kinase assay, the authors show that influenza NP is phosphorylated by ERK2. To decouple oligomerization from phosphorylation, they introduced a mutation that prevents NP oligomerization and confirmed that NP remains an ERK2 substrate. LC-MS/MS mapped three sites—S450, S472, and S473—with a couple resides conserved across influenza A and B subtypes and lineages. The authors then probed the functional role of these modifications, presenting evidence that NP phosphorylation may facilitate nuclear export. Finally, they demonstrate that NP interacts with ERK only when ERK is in its phosphorylated (active) form and describe a multikinase complex that mediates NP phosphorylation.

**Part II – Major Issues: Key Experiments Required for Acceptance**
Please use this section to detail the key new experiments or modifications of existing experiments that should be absolutely required to validate study conclusions.

Generally, there should be no more than 3 such required experiments or major modifications for a "Major Revision" recommendation. If more than 3 experiments are necessary to validate the study conclusions, then you are encouraged to recommend "Reject".

Reviewer #4: (No Response)

Reviewer 4 raised NO MAJOR COMMENTS!

Reviewer #5: - The authors analyze the subcellular localization of the influenza NP protein, and NP variants lacking its phosphorylation sites in influenza virus-infected cells, by fluorescence microscopy, and they infer from these experiments that the ERK2-mediated, NP phosphorylation facilitates the transport of the vRNPs from the nucleus to the cytoplasm at the late stages of the infection (Figure 4C). From my point of view, this is an overstated conclusion that needs further support, by, for example, analyzing the nuclear and cytoplasmic localization of viral genomic RNAs, that form part of the vRNPs. From the experiments shown in Figures 4A, 4B (in cells overexpressing the NP variants) and 4C (in infected cells), they should only conclude that the NP phosphorylation affects its subcellular localization at late time points.

Response: We concur with reviewer’s concern! To address this issue we executed two major experiments as mentioned below:

1. To establish that phospho-null mutations not only alter NP’s nuclear-cytoplasmic localization but also interfere with the nuclear export of viral RNP complexes, we infected cells with either NP-WT or NP-S450A/ S473A double mutant viruses. Nuclear-cytoplasmic distribution of RNPs were monitored at 2 and 8 hpi by staining the cells with NP and PA (Fig 4C) or NP and PB2 (Fig S6A) specific antibodies. The double mutant virus showed significant retention of all the three RNP associated proteins (NP, PB2 and PA) in the nucleus at 8 hpi.
2. Additionally, we executed nuclear cytoplasmic fractionation of cells infected with WT or double mutant viruses, harvested at 2 and 8 hpi. Relative abundance of viral genomic RNA (vRNA) and GAPDH in the respective sub-cellular compartments were analysed using Real-time PCR (qRT-PCR). As evidenced in Fig S6B, vRNAs of the WT viruses were majorly localised into the nucleus at 2 hpi and redistributed to the cytoplasm at 8hpi. In contrast, vRNAs of the double mutant virus mostly retained within the nucleus throughout the time course of infection. GAPDH mRNA prevalently localised in the cytoplasm irrespective of infection, thus validating the sub cellular fractionation procedure.

The data is included in Figure 4 and supporting figure S6 and discussed in the results (Line no: 321-335) and method (Line no: 911-933) sections of the revised manuscript. We hope that these data together will be sufficient to establish that ERK2 mediated NP phosphorylation is critical in promoting vRNP nuclear export.

- The levels of phospho-ERK should have been tested in influenza-infected cells, to confirm that ERK2 is activated in infected cells, and to show the activation kinetics at different times post-infection

Response: As per reviewer’s suggestion, we monitored activation kinetics of ERK2 in influenza A/ WSN/1933 (H1N1) virus infected A549 cells (Fig S8). The data demonstrates a biphasic activation of ERK2 at early (1-2 hpi) and late (6-8 hpi) stages of infection. The data is included in figure S8 and discussed in the result section (Line no: 419-421).

Reviewer #6: As a revised manuscript, the authors provide a large collection of datasets with figures within figures. Please take a concise effort to determine what data should be kept in the main figures and what data could be illustrated in supplementary figures.

Response: To address reviewer’s concern, we have extensively reorganised the figures and a number of data sets are now moved to supporting document.

Refrain from the use of language such as “severely restricted” when describing a virus that still can reach titers of >10^4 pfu/ml.

Response: As per the suggestion, line 129 and line 534 has been modified to remove “severely attenuated”.

**Part III – Minor Issues: Editorial and Data Presentation Modifications**
Please use this section for editorial suggestions as well as relatively minor modifications of existing data that would enhance clarity.

Reviewer #4: (No Response)

Reviewer #5: - According to my point of view, it is not clear the conclusion indicating that “Co-expression of PKCα-CAT, resulted in moderate hyperphosphorylation of NP, which gets significantly boosted in presence of ERK2, in a dose dependent manner” (lines 153-154). For example, if we compare ERK2 expression in lanes 4 and 5, the expression of ERK2 is clearly higher in lane 5 (Figure 1D), whereas the NP phosphorylation levels are similar.

Response: This statement has been modified (Line no: 155-157).

- Why the authors did not test if ERK1 participates on NP phosphorylation? This point should be at least discussed

Response: The choice of ERK2 primarily driven by the previous report [1] suggesting ERK2’s involvement in NP phosphorylation. The ERK1 and ERK2 has 85% sequence identity and shows redundant functions as well. Thus the possibility of involvement of ERK1 in this signalling event cannot be excluded. This issue has been addressed by adding additional statements in the result (Line no: 143-146) and discussion (Line no: 593-595) sections. We hope this will address reviewer’s concern.

- In Figure 1H, to what protein corresponds the lower band detected after NP immunoprecipitation (in the NP-PO3S blot), as this lower band is detected both in non-infected and infected cells?

Response: We thank reviewer for raising this issue. This is potentially a non-specific host protein which might get phosphorylated by ERK2-AS and non-specifically got immunoprecipitated. A statement related to this is included in the figure legend of Figure 1F (Line no: 1171).

- Which is the antibody used to detect phospho-NP in figure 2B? While in the presence of ERK-2AS and the ATP analogue, an antibody specific for thiophosphate ester seems to have been used, in the presence of regular ATP, this antibody should not detect any band, according to my understanding. However, in lanes 2 and 3 (Figure 2B), in which the regular ATP was used for the reaction, clear phospho-NP bands are observed. This result should be further explained.

Response: The in vitro kinase assay in Figure 2B was performed either using γ-thio ATP (lane 1-3) or γ-thio ATP analogue (lane 4-6). Subsequently, the product was alkylated using PNBM followed by detection using thiophosphate ester antibody. This is why, in lane 2 and 3, both ERK2 and ERK2-AS could conjugate the γ-thio ATP to NP-E339A while in lane 5 and 6, only ERK2-AS could utilize the γ-thio ATP analogue. We modified the result section (Line no: 201-206), figure 2B and figure legend (Line: 1177) to make it more reader friendly.

- In line 219, the words “to this end” make no sense to me. The authors should better clarify why they mutate the S450, T472 and S473 residues to phospho-null alanine, either individually or in combination, in the monomeric NP-E339A.

Response: As shown in figure 2A, the monomeric NP-E339A is a superior substrate towards ERK2 mediated phosphorylation compared to the WT NP. This is because a large number of phosphorylation sites in NP, which are situated near the oligomerization interface, remains inaccessible towards the NP specific kinases. This was also reported in our previous study where NP-E339A showed higher propensity towards PKC-δ mediated phosphorylation than the WT NP [2]. That is why, the phosphonull mutations, S450A, T472A and S473A, were introduced in NP-E339A, to nullify any potential effect of NP oligomerization upon the accessibility of the abovementioned phospho-sites towards the kinase, in this case ERK2. This justification was already incorporated at the very beginning result subsection 2 (Line no: 194-199).

Additionally, the sentence, pointed out by the reviewer has now been modified to remove “to this end” and also to reiterate the justification of using NP-E339A in this specific experiment (Line no. 223-227).

- In the virus-infected cells, the authors detect that NP is phosphorylated at residues S450, T472 and S473, however, they only generate the mutant viral strains NP-S450A, NP-S473A. Why the authors did not generate the T472A mutant virus? This point should be further explained.

Response: S450 and S473, but not the T472, phosphorylation sites were identified through MS analysis of the ERK2 mediated in vitro phosphorylation of NP, while all three of them were identified from the NP purified from the infected cells. Possibly, the T472 and S473 represent functionally redundant phosphorylation sites; hence only S473 is included in this analysis. This justification is included in the revised manuscript (Line 245-248).

-It is surprising that in cells knocked-down for PCK⍺ (the shPKC⍺-1 cells), the expression of PKC⍺ is not much higher than in the PCK⍺-2 and PKC⍺1+2 cells, and despite this, viral titers at 12 h vary greatly among the shPKC⍺-1 cells and the shPKC⍺-2 cells or the shPKC⍺-1+2 cells. Do the authors have an explanation for this?

Response: The shPKCα-2 showed marginally higher knockdown efficacy than the shPKCα-1 cell line. As per our experience with these knockdown cell lines, the shPKCα-2 always showed more restriction towards virus replication (compared to the shNTC) than the shPKCα-1 cell line. This suggests that even a marginally higher expression of the PKC⍺ can boost virus replication to a significant extent. Possibly this is why, the shPKCα-1cell line, which shows slightly lesser efficiency of knockdown, supports virus replication to a higher extent than shPKCα-2 cell line, at least during the initial cycles of replication (as observed at 12hpi). However, at 24 hpi significant reduction in viral titre was observed in both the cell lines. A modified statement explaining this observation is incorporated in the specific portion of the result section (Line no: 499-503).

- In the co-IP assays, I miss a control. For example, after immunoprecipitating ERK2, NP is co-immunoprecipitated in the presence of PKC⍺, however, a control in which is ERK is not expressed, but NP and PKC⍺ are expressed is needed to discard the possibility that NP binds inespecifically to the beads used for the immunoprecipitation.

Response: We thank the reviewer for raising this concern. We have repeated the experiment with requested control set and incorporated the data in the Figure S6. Result section has also been modified accordingly (Line: 353).

- Similarly, in the PLA assays (Figures 8E and 8F), I miss a control using mock-infected cells and staining the cells with the anti-NP and anti-ERK antibodies.

Response: The PLA experiment presented in Figure 8 actually contains the Mock infected set which was stained with anti-NP goat and anti-ERK-rabbit antibodies. For better clarity we have modified the Figure 8 and incorporated the detailed panel of the microscopic data (with proper labelling of the antibodies) in Figure S8.

Reviewer #6: Figure 3 B – Y-axis should start at 1.

Response: Figure 3B is modified accordingly.

PLOS authors have the option to publish the peer review history of their article ([what does this mean?](https://track.editorialmanager.com/CL0/https:%2F%2Fjournals.plos.org%2Fplospathogens%2Fs%2Feditorial-and-peer-review-process%23loc-peer-review-history/1/010f0199e9d15ba3-61f99788-859e-4481-9aa0-c66ef10795b9-000000/Uof4Mw2qpXwh3Ps0qK-mRq3HQ00Ev2gTdGoM4_Y1AzY=233)). If published, this will include your full peer review and any attached files.


If you choose “no”, your identity will remain anonymous but your review may still be made public.


**Do you want your identity to be public for this peer review?** For information about this choice, including consent withdrawal, please see our [Privacy Policy](https://track.editorialmanager.com/CL0/https:%2F%2Fwww.plos.org%2Fprivacy-policy/1/010f0199e9d15ba3-61f99788-859e-4481-9aa0-c66ef10795b9-000000/H-idA4ANYem8IGNeDX7rZbAcZkT2nz_lqwBbm4V67TE=233).

Reviewer #4: No

Reviewer #5: No

Reviewer #6: No

**Figure resubmission:**

While revising your submission, we strongly recommend that you use PLOS’s NAAS tool (<https://ngplosjournals.pagemajik.ai/artanalysis>) to test your figure files. NAAS can convert your figure files to the TIFF file type and meet basic requirements (such as print size, resolution), or provide you with a report on issues that do not meet our requirements and that NAAS cannot fix.

After uploading your figures to PLOS’s NAAS tool - <https://ngplosjournals.pagemajik.ai/artanalysis>, NAAS will process the files provided and display the results in the "Uploaded Files" section of the page as the processing is complete. If the uploaded figures meet our requirements (or NAAS is able to fix the files to meet our requirements), the figure will be marked as "fixed" above. If NAAS is unable to fix the files, a red "failed" label will appear above. When NAAS has confirmed that the figure files meet our requirements, please download the file via the download option, and include these NAAS processed figure files when submitting your revised manuscript

**Reproducibility:**

To enhance the reproducibility of your results, we recommend that authors of applicable studies deposit laboratory protocols in [protocols.io](http://protocols.io/), where a protocol can be assigned its own identifier (DOI) such that it can be cited independently in the future. Additionally, PLOS ONE offers an option to publish peer-reviewed clinical study protocols. Read more information on sharing protocols at <https://plos.org/protocols?utm_medium=editorial-email&utm_source=authorletters&utm_campaign=protocols>

 References:

1.	Pleschka S, Wolff T, Ehrhardt C, Hobom G, Planz O, Rapp UR, et al. Influenza virus propagation is impaired by inhibition of the Raf / MEK / ERK signalling cascade. 2001;3.

2.	Mondal A, Dawson AR, Potts GK, Freiberger EC, Baker SF, Moser LA, et al. Influenza virus recruits host protein kinase C to control assembly and activity of its replication machinery. Elife. 2017;6: 1–23. doi:10.7554/eLife.26910.001
